# Reappraisal of metabolic dysfunction in neurodegeneration: *Focus on mitochondrial function and calcium signaling*

**DOI:** 10.1186/s40478-021-01224-4

**Published:** 2021-07-07

**Authors:** Pooja Jadiya, Joanne F. Garbincius, John W. Elrod

**Affiliations:** Center for Translational Medicine, Lewis Katz School of Medicine at Temple University, 3500 N Broad St, MERB 949, Philadelphia, PA 19140 USA

**Keywords:** Mitochondria, Metabolism, Calcium, Neurodegeneration, Alzheimer’s disease, Parkinson's disease, Huntington's disease

## Abstract

The cellular and molecular mechanisms that drive neurodegeneration remain poorly defined. Recent clinical trial failures, difficult diagnosis, uncertain etiology, and lack of curative therapies prompted us to re-examine other hypotheses of neurodegenerative pathogenesis. Recent reports establish that mitochondrial and calcium dysregulation occur early in many neurodegenerative diseases (NDDs), including Alzheimer's disease, Parkinson’s disease, Huntington's disease, and others. However, causal molecular evidence of mitochondrial and metabolic contributions to pathogenesis remains insufficient. Here we summarize the data supporting the hypothesis that mitochondrial and metabolic dysfunction result from diverse etiologies of neuropathology. We provide a current and comprehensive review of the literature and interpret that defective mitochondrial metabolism is upstream and primary to protein aggregation and other dogmatic hypotheses of NDDs. Finally, we identify gaps in knowledge and propose therapeutic modulation of _m_Ca^2+^ exchange and mitochondrial function to alleviate metabolic impairments and treat NDDs.

## Introduction

The brain consumes 20% of the body’s ATP at rest, although it accounts for only 2% of body mass [1]. The high-energy requirements of the brain support neurotransmission, action potential firing, synapse development, maintenance of brain cells, neuronal plasticity, and cellular activities required for learning and memory [2, 3]. In neurons, most of the energy is consumed for synaptic transmission. Action potential signaling represents the second-largest metabolic need, and it is estimated that ~ 400–800 million ATP molecules are used to reestablish the electrochemical gradient (Na^+^ out, K^+^ in, at the plasma membrane) after production of the single action potential [4]. The energetic demand of neurons results in a substantial dependence on mitochondria for ATP production through oxidative phosphorylation (OxPhos) [4]. Any dysfunction in mitochondria can lessen the energetic capacity of OxPhos and may elicit a metabolic switch from OxPhos to glycolysis (Warburg-like effect) as a compensatory attempt to maintain cellular ATP in the context of neurodegenerative stress [5, 6]. However, a long-term OxPhos-to-glycolysis shift can result in a bioenergetic crisis and make neurons more vulnerable to oxidative stress and neuronal cell death [7, 8].

Neurodegenerative diseases (NDDs) are characterized by numerous cellular features, including the loss of neurons, neuronal dysfunction in specific brain regions, aggregation of distinct protein(s), impaired protein clearance, mitochondrial dysfunction, oxidative stress, neuroinflammation, axonal transport defects and cell death. The myriad of cellular pathologies suggest that there are common/central molecular mechanisms driving NDDs [9, 10]. In addition to ATP production, the mitochondrion is an epicenter of many metabolic pathways and important cellular functions, including the fine-tuning of intracellular calcium (_i_Ca^2+^) signaling, regulation of cell death, lipid synthesis, ROS signaling, and cellular quality control [11]. Disruption in mitochondrial function and metabolism appears to underlie several NDDs such as Alzheimer’s disease (AD), Parkinson’s disease (PD), Huntington’s disease (HD), and others [12, 13]. At present, most therapies for NDDs provide only symptomatic relief, and there remain no drugs to inhibit neurodegeneration [14–16]. Mitochondrial alterations/impaired brain energetics are thought to present in the asymptomatic stage of disease prior to the onset of clinical symptoms [14, 17, 18]. This supports the notion that mitochondrial metabolic defects may be drivers or even initiators of the neurodegenerative process. In addition, several therapeutics that improve mitochondrial function have been reported to be efficacious in NDD models [19–21].

Mitochondrial calcium (_m_Ca^2+^) is a critical regulator of mitochondrial function. In the matrix, _m_Ca^2+^ tightly regulates TCA cycle activity and augments metabolic output. However, an excess of _m_Ca^2+^ can impair mitochondrial respiration, enhance reactive oxygen species (ROS) production and activate cell death [22]. Here, we hypothesize that dysfunction in _m_Ca^2+^ is an early common cellular event that impairs mitochondrial metabolism and drives and exacerbates neuropathology. Defining the molecular basis of mitochondrial function and metabolism in NDDs will help define novel cellular events and pathways and their temporal occurrence in NDD progression to identify new therapeutic targets for various neurological conditions. Here, we review recent advancements in our understanding of the essential role of mitochondrial metabolism and discuss how impaired _m_Ca^2+^ signaling may be causal and central in neurodegeneration.

## Evidence for impaired mitochondrial metabolism in NDDs

Strategies to combat NDDs have generally been unsuccessful and are focused on reducing symptoms and disease modification. Both clinical and experimental studies suggest that impaired energy metabolism correlates with various neurological deficits, highlighting new therapeutic opportunities [14]. Here we outline various mitochondrial metabolic defects that are strongly linked to the progression of neurodegeneration.

### Alzheimer’s disease (AD)

AD is the most common form of dementia and is characterized by irreversible memory loss due to neuronal dysfunction, dysconnectivity, and cell death. Familial AD (FAD) is caused by pathogenic mutations in amyloid precursor protein (APP) or presenilin (PS1 and PS2) that lead to overproduction, improper cleavage, and the accumulation of amyloid-beta (Aβ). Prognostic disease phenotypes are associated with the formation of Aβ plaques, neurofibrillary tangles (NFTs, consisting of the microtubule protein tau), synaptic failure, reduced synthesis of the neurotransmitter acetylcholine, and chronic inflammation [9]. Most therapeutic strategies have been focused on Aβ metabolism and clearance due to extensive preclinical and clinical data in support of a causal role in AD progression [23, 24]. According to the “amyloid cascade hypothesis,” Aβ aggregation can initiate a series of events, including tau pathology, oxidative stress, inflammation, neuronal calcium (Ca^2+^) dysregulation, and metabolic alterations, which culminate in neuronal cell loss and AD pathogenesis [25]. However, this hypothesis does not fully explain the etiology of sporadic forms of AD (SAD) that account for 90–95% of AD-associated dementia.

An alternative hypothesis is that the microtubule-associated protein tau becomes hyperphosphorylated, resulting in axonal transport defects of organelles (including mitochondria), synaptic dysfunction, and cell death [26]. In cortical brain tissue from AD patients and mouse models, tau is reported to interact with mitochondrial transporters and complexes, resulting in mitochondrial dysfunction and AD pathology [27, 28]. However, there appears to be a limited correlation between the severity of cognitive decline and amyloid or tau plaque formation [29, 30], suggesting Aβ/tau metabolism and processing may not be the cause, or at least the singular cause, of disease. Consistent with previous studies, RNA-sequencing data from AD patients also suggest that Aβ and tau accumulation may not be mediators of the disease [31, 32]. Also, clinical trials of therapies targeting Aβ/tau production, metabolism, and clearance have universally shown little efficacy making it likely that other proximal mechanisms of AD pathogenesis exist [33, 34].

Mitochondrial dysfunction appears to be a primary occurrence in AD that precedes Aβ deposition, synaptic degeneration, and NFTs formation. In support of this concept, cytoplasmic hybrid cells (cybrids) generated from platelet mitochondria of SAD patients were reported to have a deficiency in complex I and complex IV of the electron transport chain (ETC), reduced mitochondrial membrane potential (Δψm), altered mitochondrial morphology, increased Aβ generation and tau oligomerization (reviewed in [35]). Transmission electron microscopy (TEM) showed smaller mitochondria with altered cristae structure and a decrease in mitochondrial content both in AD mice and patients [36–38]. Furthermore, fibroblasts derived from SAD patients also showed impaired mitochondrial dynamics, bioenergetics, and Ca^2+^ dysregulation [17, 39]. This change in mitochondria morphology in AD may be due to a shift in the mitochondrial fission/fusion balance and a decrease in biogenesis [40].

Importantly, experimental evidence suggests that bioenergetic alterations in AD precede the formation of Aβ plaques [41]. Data supporting metabolic deficits in AD were first published in the early 1980s from 2-[^18^F] fluoro-2-deoxy-D-glucose (FDG) positron emission tomography (PET) studies, which showed reduced glucose metabolism in the parietal, temporal and frontal cortex of AD patients [42–44]. Postmortem brain tissue isolated from AD patients displays reduced mitochondrial metabolic enzyme activity for pyruvate dehydrogenase (PDH) [45, 46], alpha-ketoglutarate dehydrogenase (α-KGDH) [46], isocitrate dehydrogenase (ICDH) [47], and complex IV or cytochrome-c-oxidase (COX) [48–50]. In addition, succinate dehydrogenase (SDH) and malate dehydrogenase (MDH) activity are increased in AD patient's brains [51]. Microarray data [52] and bioinformatics analysis of four transcriptome datasets [53] suggests a significant downregulation in nuclear-encoded OxPhos genes in the hippocampus of AD patients. More recent data confirm impaired ATP synthase activity due to loss of the oligomycin sensitive conferring protein subunit in the brain of FAD and SAD patients [54].

Diminished PDH function, as noted in AD, limits the shuttling of pyruvate into the TCA cycle, causing pyruvate accumulation and favoring anaerobic metabolism. Anaerobic metabolism leads to the production of lactic acid and further reduces acetyl-CoA availability, which subsequently decreases OxPhos. These observations suggest a metabolic shift from OxPhos to glycolysis may occur with AD progression. This shift is perhaps a compensatory response to enhance energy production through glycolysis, which is noteworthy in the context of mitochondrial dysfunction [5, 6]. Interestingly, PDH, α-KGDH, and ICDH activity are all reported to be calcium-controlled, suggesting a clear link between _m_Ca^2+^ levels and AD pathogenesis, which will be discussed in the upcoming section. A recent study also indicates that reduced mitochondrial pyruvate uptake in FAD-PS2-expressing cells may elicit impairments in bioenergetics and mitochondrial ATP synthesis [13]. The mechanism for defective mitochondrial pyruvate flux is associated with the hyper-activation of glycogen-synthase-kinase-3β (GSK3β), which decreases hexokinase 1 association with mitochondria and destabilizes the mitochondrial pyruvate carrier complexes [13]. Similarly, α-KGDH is sensitive to oxidative stress, and its reduced activity in PS1 mutant (M146L) fibroblasts suggests a possible mechanism for ROS-dependent metabolic deficiencies [18, 55]. Oxidative stress, as seen in AD brains [56], is reported to increase the expression of *SDHA* (one of the four nuclear-encoded subunits of complex II, SDH) [57, 58], and the activity of MDH [59]. In summation, alterations in key metabolic enzymes may compromise the neurons’ ability to generate ATP via OxPhos and be an early driver of cellular stress in AD.

Beyond energetic compromise, diminished acetyl-CoA supply caused either by a reduction in glucose metabolism or by reduced PDH activity impairs the synthesis of the neurotransmitter acetylcholine (ACh). ACh is generated from choline and acetyl-CoA by choline acetyltransferase. After synthesis, ACh is transported via an ATP-consuming process and stored in synaptic vesicles [60]. The loss of ACh synthesis in AD results in defective cholinergic neurotransmission [61, 62]. This provides another tangible link between energetic compromise and neuronal dysfunction in AD.

Several of the mitochondrial dehydrogenases mentioned above (PDH, α-KGDH, and ICDH) are known to be regulated by the Ca^2+^ concentration within the mitochondrial matrix [63–65]. The reactions catalyzed by the Ca^2+^-regulated mitochondrial dehydrogenases are rate-limiting steps in the TCA cycle, and therefore free-Ca^2+^ content in the mitochondrial matrix is a major regulator of metabolic output. PDH activity increases upon dephosphorylation of its E1α subunit, which is mediated by the Ca^2+^-sensitive phosphatase (PDP1) [64]. In neurons, Ca^2+^ influx through voltage-dependent Ca^2+^ channels is required for the fusion of synaptic vesicles with the plasma membrane and release of neurotransmitters at the synaptic cleft [66, 67]. Neuronal communication through synaptic transmission is an energy-demanding process, and mitochondria have a critical role in this process by providing ATP (via OxPhos) and by buffering synaptic Ca^2+^/_i_Ca^2+^ to modulate neurotransmitter release [68]. The efficient regulation and buffering of _i_Ca^2+^ is critical to prevent neuronal excitotoxicity. Mitochondria and the endoplasmic reticulum (ER) both are significant modulators of _i_Ca^2+^ signaling and the role of ER in neuronal _i_Ca^2+^ buffering is well known [69, 70]. However, our understanding of _m_Ca^2+^ buffering in neurons is limited and evolving. Ca^2+^ enters the mitochondrial matrix through the mitochondrial calcium uniporter channel (mtCU) [71, 72] and is extruded via the mitochondrial Na^+^/Ca^2+^ exchanger (NCLX) [73, 74]. Any dysfunction in _m_Ca^2+^ exchange or matrix buffering capacity can lead to impairments in mitochondrial Ca^2+^ homeostasis resulting in _m_Ca^2+^ overload, oxidative stress, metabolic dysfunction, and cell death that can cause or precede AD-pathology [75–78]. We and others have reported that mitochondrial and metabolic dysfunction is a primary contributor to AD pathogenesis, with dysfunction observable before the appearance of Aβ aggregates and NFTs [18, 77, 79, 80]. We found alterations in the expression of _m_Ca^2+^ handling genes in samples isolated from the brains of SAD patients post-mortem and in the triple transgenic mouse model of AD (3xTg-AD) prior to observable AD pathology [77]. Our observations suggest _m_Ca^2+^ overload caused by an age-dependent remodeling of _m_Ca^2+^ exchange machinery contributes to the progression of AD by promoting metabolic and mitochondrial dysfunction. We also found a decrease in OxPhos capacity in APPswe cell lines (K670N, M671L Swedish mutation), providing further evidence of impaired mitochondrial metabolism in AD [77]. Importantly, the genetic rescue of neuronal _m_Ca^2+^ efflux capacity by expression of NCLX in 3xTg-AD mice was sufficient to block age-dependent AD-like pathology [77]. Employing quantitative comparative proteomics strategies in AD mice, other groups have reported significant alterations in the mitochondrial proteome, including the citric acid cycle, OxPhos, pyruvate metabolism, glycolysis, oxidative stress, ion transport, apoptosis, and mitochondrial protein synthesis well before the onset of the AD phenotype [79–81]. Further evidence of _m_Ca^2+^ dysregulation is from metabolomics in an Aβ-transgenic *C. elegans* model (GRU102), wherein the authors showed a reduction in TCA cycle flux before the appearance of significant Aβ deposition, with the greatest reduction observed in α-KGDH activity. Knockdown of α-KGDH in control worms elicited reductions in both basal and maximal respiration like that observed in the AD worm model [18]. These observations suggest that reduced α-KGDH activity alone is sufficient to recapitulate the metabolic deficits observed in AD and is in line with a study by Yao et al. [46] wherein 3-month old 3xTg-AD mice were found to have reduced mitochondrial respiration and PDH activity, coupled with increased ROS generation [46]. Altogether, these data indicate that _m_Ca^2+^ dysregulation is likely an early event in AD.

Mitochondria are highly dynamic, and exhibit cell type-specific metabolism in the brain [37, 82]. Axonal mitochondria appear small and sparse whereas dendritic mitochondria are elongated and more densely packed [82]. To ensure appropriate energy supply, especially in distal regions of the axons, mitochondria must be properly positioned. Indeed, mitochondria undergo bi-directional axonal transport including anterograde transport (from cell body to axon) and retrograde transport (from axon to cell body) [83, 84]. Axonal transport is mediated by ATP‐hydrolyzing motor proteins (kinesin‐I for anterograde and dynein for retrograde) to move cargo along microtubule tracks [85] and defects in transport seem to present before evident AD hallmarks [86, 87]. Defects in anterograde transport result in an insufficient supply of ATP at the synapse, resulting in synaptic starvation and dysfunction, an early pathological feature of AD [36]. Similarly, defective retrograde transport can lead to the accumulation of damaged mitochondria, which can compromise mitochondrial quality control mechanisms, which is also noted to occur in AD [88]. Recently, data from the APP-PS1 mouse model showed a reduction in neuronal mitochondria density around amyloid plaques, suggesting impaired mitochondrial transport and/or quality control in AD [37]. Further, several studies indicate that axonal transport of AD-associated proteins becomes defective early in disease progression, resulting in the accumulation of toxic cargo which can elicit protein aggregation, axonal swellings, and neuronal dysfunction [36, 87]. The mechanisms regulating axonal transport are not completely understood but some studies suggest that it is mediated by the interaction of kinesin motor protein with the mitochondrial adaptor proteins, Miro and Milton (known as trafficking kinesin protein (TRAK) family) [89]. Miro is a GTPase with two Ca^2+^ binding EF-hand domains and is localized to the outer mitochondrial membrane (OMM) and has an essential role in Ca^2+^-dependent regulation of mitochondrial transport. Intriguingly, Miro1 may also serve as a cytoplasmic Ca^2+^ sensor and may increase _m_Ca^2+^ uptake via interaction with MCU’s N-terminal domain [90, 91]. An increase in _m_Ca^2+^ has been shown to inhibit mitochondrial axonal transport and blocking _m_Ca^2+^ influx into mitochondria by direct MCU inhibition enhances mitochondrial trafficking in axons [90].

**Fig. 1:**
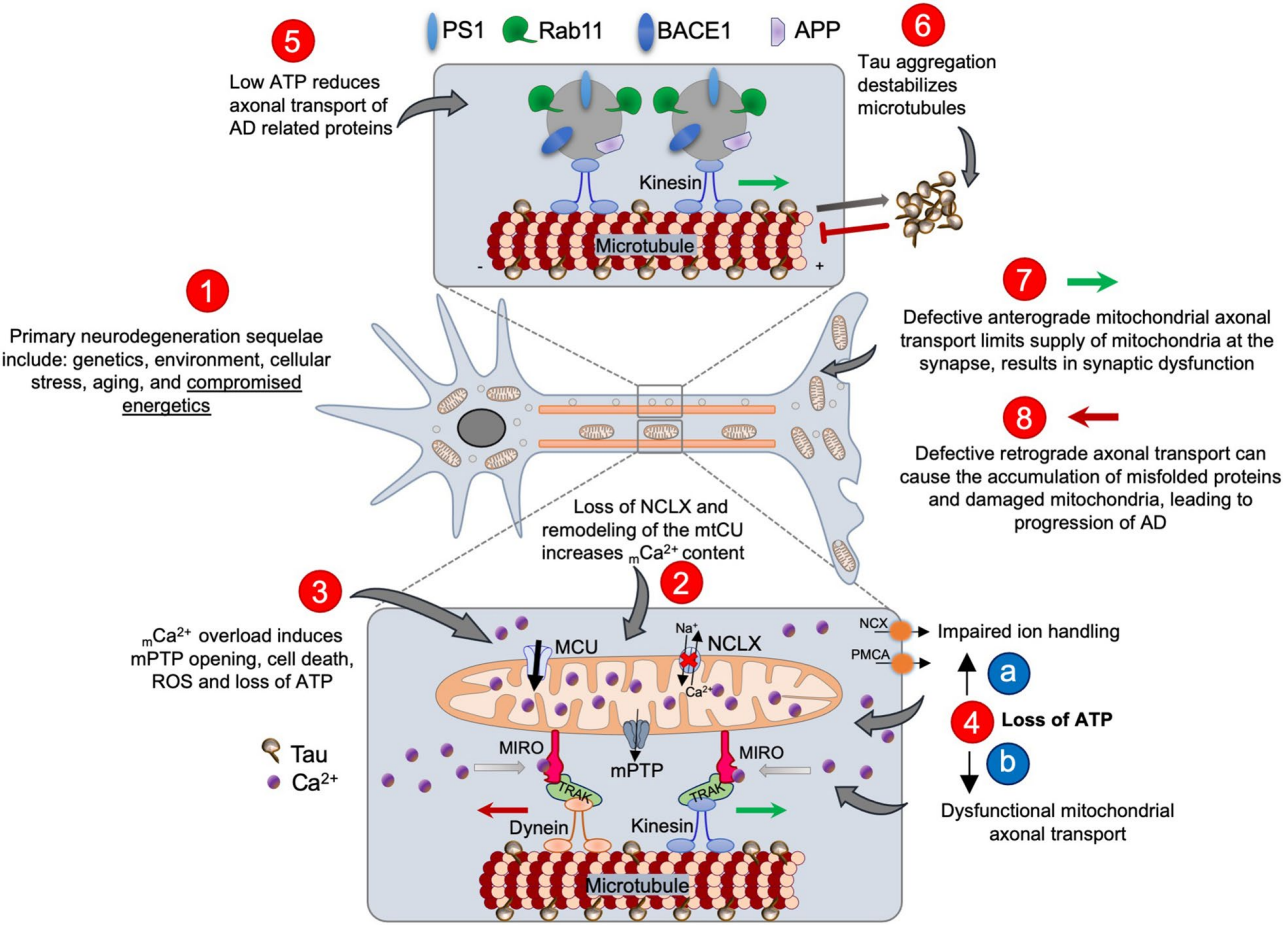
Hypothetical mechanisms of _m_Ca^2+^ overload-induced cellular dysfunction in AD progression. Loss of NCLX and remodeling of the mtCU causes _m_Ca^2+^ overload that leads to mPTP opening, loss of ATP, and interrupted axonal transport, resulting in AD progression

While multiple molecular mechanisms likely contribute to AD pathogenesis, the data suggest that neuronal _m_Ca^2+^ overload is a primary mediator of AD progression, causing impaired mitochondrial metabolism and ATP production, mitochondrial transport, and increased mitochondrial permeability transition pore (mPTP) opening (Fig. 1). This in turn results in loss of synaptic function, amyloid deposition, tau pathology, and cell death.

### Parkinson’s disease (PD)

PD is the second most common NDD afflicing ~ 1% of the population above 60 years of age [92]. It is clinically characterized by both motor dysfunction such as tremor (involuntary shaking), bradykinesia (slowness of movements), rigidity (resistance to movement), and akinesia, as well as non-motor disturbances such as depression, anxiety, fatigue, and dementia. These symptoms are caused by a diminishment of the neurotransmitter dopamine due to degeneration of dopaminergic neurons in the pars compacta of the substantia nigra in the midbrain and the deposition of intraneuronal proteinaceous inclusions known as Lewy bodies that are mainly composed of α-synuclein [93]. Most PD cases are sporadic with no known singular cause. Familial PD is associated with mutations in many genes including: *SNCA* (α-synuclein) [94], *PRKN* (parkin) [95], *PARK7* (DJ-1) [96]*, LRRK2* (leucine-rich repeat kinase 2) [97]*,* and *PINK1* (phosphatase and tensin homologue (PTEN)-induced kinase 1) [98]. Studies suggest that homozygous mutations in *Parkin* are the most common cause of juvenile PD, but their role in idiopathic PD is unclear. Mutations in *Parkin* are not reliably associated with Lewy body pathology. Post-mortem examination of patients with *Parkin* mutations shows a clinical phenotype of dopaminergic neuronal loss and gliosis but lacking Lewy body pathology. However, this remains controversial as a few case reports demonstrate the presence of Lewy pathology in patients with *Parkin* mutations. Further studies are needed to define if parkin and Lewy body pathology are in linear pathways (reviewed in [99]).

Drug therapy for PD is limited and is primarily focused on enhancing dopamine levels via administration of l-3,4-dihydroxyphenylalanine (L-DOPA or Levodopa), which is metabolized to dopamine after crossing the blood–brain barrier [100, 101]. However, this therapy is only effective in the early stages of disease, and provides symptomatic relief with many adverse side effects, and is insufficient to block the progression of PD [15, 102], suggest a crucial need for new, effective therapies [103, 104]. Although the exact mechanisms of PD pathogenesis are not clear, many possible molecular events have been proposed to contribute to this process.

Mitochondrial dysfunction and impaired cellular bioenergetics have emerged as likely mechanisms driving PD pathogenesis in several studies [105, 106]. Dopaminergic neurons consume ~ 20-times more energy as compared to other neurons because of their anatomical structure (extensive long and branched axons), greater number of transmitter release sites, and their pacemaking activity [107]. The high-energetic demand of dopaminergic neurons makes them more susceptible to mitochondrial dysfunction and eventually to cell death in comparison to other neuronal cells [108, 109]. Defects in mitochondrial respiration are supported by findings of reduced glucose utilization in PD patients [110], as well as reduced pyruvate oxidation in fibroblasts derived from PD patients [111], which suggest reduced acetyl-CoA entry into the TCA cycle. The first study showing that defects in mitochondrial respiration may be causal in PD came in the early 1980s. In this study, experimental inhibition of complex I (NADH-ubiquinone reductase) of the ETC was sufficient to cause parkinsonism [112, 113]. This is consistently supported by observations of a profound reduction in ETC activity, mostly complex I, in the substantia nigra, platelets, and skeletal muscle of PD patients [114]. Furthermore, inhibitors of complex I, such as MPP^+^ (1-methyl-4-phenylpyridinium), 6-hydroxydopamine, rotenone and annonacin all elicit PD-like phenotypes, suggesting that mitochondrial dysfunction is sufficient to promote neuronal dysfunction in PD [115–117]. Complex I is a key entry point for electrons into the respiratory chain and is responsible for ~ 40% of mitochondrial ATP production [118, 119]. In addition to complex I, a reduction in complex II and III activity and the mitochondrial DNA (mtDNA) transcription factor, TFAM, has also been reported in PD patients [120–122]. Reduced ETC capacity in PD may cause a significant reduction in ATP [123] resulting in a cellular energy crisis that can impact various processes including: (1) ATP-dependent proton pumps that drive vesicular accumulation of dopamine [124, 125]; (2) axonal transport of cargo [126]; (3) mitochondrial dynamics (fusion, fission, turnover, biogenesis and transport) [127, 128]; and (4) ATP-dependent protein degradation systems (e.g. ubiquitin–proteasome and autophagy) [129, 130]. In addition, complex I and III deficiency in PD is linked with increased production of free radicals that further impair mitochondria function, drive protein aggregation and culminate in cell death [131–133]. Dopamine is very unstable and sequestered inside synaptic vesicles via the ATP-dependent vesicular monoamine transporter. If not sequestered, it is metabolized by monoamine oxidase to the toxic dopamine metabolite 3,4 dihydroxyphenylacetaldehyde, which contributes to oxidative stress, mPTP opening, and dopaminergic neuronal cell death [134]. Over the past decades, many PD-associated genetic mutations have been found to elicit changes in mitochondrial function and metabolism, supporting the notion that mitochondrial dysfunction is implicated in neuronal cell loss associated with familial PD and vice versa [98]. Mutant α-synuclein localizes to the inner mitochondrial membrane [135] and inhibits complex I activity, and promotes oxidative stress [136]. The interaction of α-synuclein with mitochondria can result in cytochrome c release, increased _m_Ca^2+^ levels, changes in mitochondrial morphology, and a decline in mitochondrial respiration. α-synuclein-mitochondrial interplay may also inhibit autophagic clearance and increase its aggregation propensity (reviewed in [137]).

A recent study suggested that mitochondrial impairments occur with Lewy body formation [138]. Furthermore, loss of function mutations in DJ-1 caused impairments in OxPhos, and complex I assembly resulting in decreased ATP production, oxidative stress, and increased glycolysis [139, 140]. These findings raise the possibility that mitochondrial dysfunction is causal in maladaptive protein aggregation. Furthermore, Parkin, as an E3 ubiquitin ligase, is directly involved in the proteasomal degradation of protein aggregates. It localizes to mitochondria and prevents cytochrome c release, mitochondrial swelling, and the accumulation of α-synuclein, which may protect dopaminergic neurons from mitochondrial and neuronal dysfunction [141–143].

Parkin and PINK1 are required for mitochondrial quality control [144, 145]; thus, loss of Parkin/PINK1 function is hypothesized to cause the accumulation of dysfunctional mitochondria that impair neuronal function. Previous work revealed that PINK1 deficient neurons display reduced NCLX-dependent _m_Ca^2+^ efflux resulting in matrix Ca^2+^ overload and subsequent mPTP opening, mitochondrial oxidative stress, lower Δψ_m_, and diminished OxPhos [146]. Furthermore, fibroblasts derived from patients with *PINK1* mutations also exhibited impaired mitochondrial metabolism, low Δψm, and low respiration, which was linked to reduced substrate availability [147]. In addition, the activation of NCLX via protein kinase A (PKA)-dependent phosphorylation of serine 258, a putative NCLX regulatory site, increases _m_Ca^2+^ efflux and protects PINK-1 deficient neurons from mitochondrial dysfunction and cell death [148]. This paradigm fits with previous reports where _m_Ca^2+^ overload caused by increased _m_Ca^2+^ uptake (via ERK1/2-dependent upregulation of MCU) caused dendritic degeneration in a late-onset familial PD model (mutation in Leucine-Rich Repeat Kinase 2) [149], and a report of MCU overexpression eliciting excitotoxic cell death [78]. Along the same line, inhibition of MCU is protective in zebrafish models of PD [150, 151]. These findings suggest _m_Ca^2+^ overload is a contributor to PD progression.

In summary, increasing evidence supports the centrality of impaired mitochondrial function and metabolism in both sporadic and familial PD, resulting in oxidative stress, ETC dysfunction, defective mitochondrial quality control, protein aggregation, progressive cellular dysfunction, and neurodegeneration.

### Huntington's disease (HD)

HD is an autosomal-dominant neurodegenerative disease resulting from an expansion of cytosine–adenine–guanine (CAG) repeats (> 35 bp) within the coding sequence of the huntingtin gene (*HTT*). Mutant huntingtin protein (mHtt) is prone to proteolytic cleavage, misfolding, and aggregation. Clinically, HD is characterized by progressive motor, cognitive, and behavioral dysfunction largely due to the loss of γ-aminobutyric acid (GABAergic) medium spiny neurons in the striatum [152]. The energy impairment hypothesis of HD was first proposed in the early 1980s from clinical observations, which revealed deficits in brain glucose utilization and weight loss in HD patients [153, 154]. Consistently, compelling evidence from PET studies suggests decreased glucose utilization in HD brains [155, 156], suggesting a defect in metabolism. In addition, compared to a control population, pre-symptomatic HD children, with no manifest symptoms, revealed a lower body mass index suggesting energy dysregulation and impairments in anabolic growth [157].

In HD patients, many key enzymes of the TCA cycle and ETC display reduced expression, including PDH, SDH, complex II, III, and IV [158]. In addition, HD patients increase lactate production in the pre-symptomatic phase of HD, indicating a possible reduction in oxidative mitochondrial metabolism and metabolic shift from OxPhos to glycolysis [159–162]. Irreversible inhibition of SDH by chronic administration of 3-nitropropionic acid in both rodents and non-human primates elicited regional lesions in the striatum accompanied by HD-like pathology [163–165]. These results suggest that defects in key TCA cycle enzymes are sufficient to drive HD-pathology. Furthermore, treatment of an HD mouse model with coenzyme Q and creatine for energy supplementation resulted in increased longevity and improved motor function [166, 167], suggesting that improving mitochondrial function and cellular bioenergetics is a viable therapeutic approach to treat HD.

Various other changes in mitochondrial function have been reported in HD. Recently, an examination of HD patient-derived induced pluripotent stem cells (iPSCs) and differentiated neural stem cells revealed altered mitochondria morphology (round and fragmented structure), lower mitochondrial respiration, decreased ATP levels and complex III activity, activation of apoptosis, and increased glycolysis [168]. Proteomic analysis in undifferentiated human HD embryonic stem cells found a decrease in key proteins involved in the ETC before observable differences in huntingtin protein [169]. These studies suggest that mitochondrial function is impaired early in HD pathogenesis. Also, mitochondrial dysfunction is linked with glutamate-mediated excitotoxicity in HD, and this is linked to defects in _m_Ca^2+^ homeostasis. Studies indicate early abnormalities in _m_Ca^2+^ that contribute to HD pathology [170]. For example, mitochondria from HD patients have an increased probability of mPTP opening, mitochondrial swelling, oxidative stress, and _m_Ca^2+^ overload [170, 171]. As in other NDDs, impaired axonal transport is also reported in HD [172] and may be caused by mitochondrial dysfunction and impaired ATP production. Overall, these findings support a prominent role for mitochondrial and metabolic defects in HD pathogenesis.

**Fig. 2:**
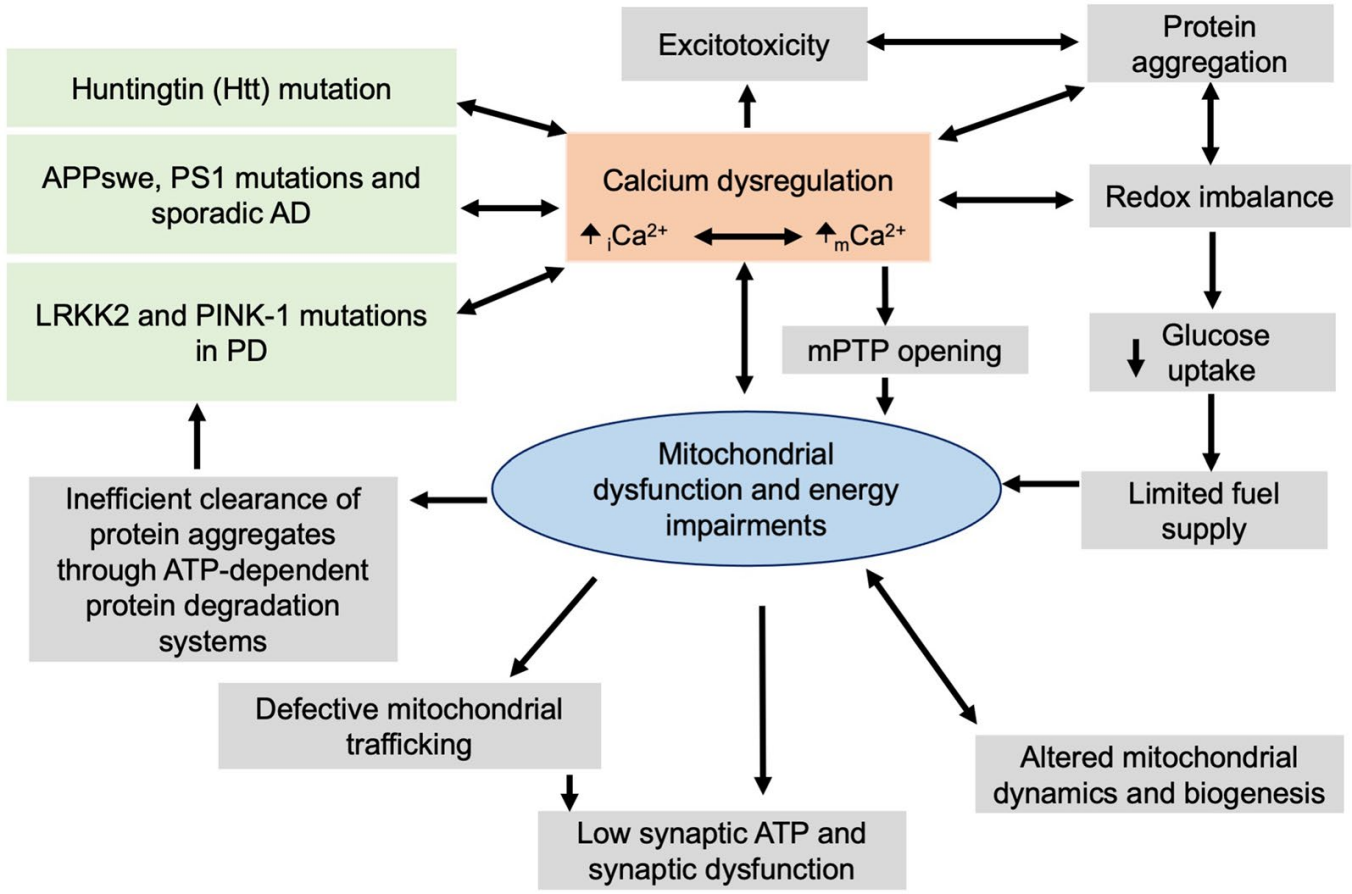
Mitochondrial and metabolic dysfunction in neurodegeneration. Mitochondrial dysfunction and energy impairments are central events in neurodegeneration

Altogether, numerous studies support that mitochondrial dysfunction and energy impairments occur before overt pathological symptoms and appear to be central in driving the progression of various NDDs. We hypothesize that metabolic and mitochondrial dysfunction is a result of _i_Ca^2+^ dysfunction and remodeling of the _m_Ca^2+^ exchange machinery, which, although initially meant to be compensatory, causes a series of events that culminate in neurodegeneration (Fig. 2).

## Molecular mechanisms of altered metabolism in NDDs

**Fig. 3:**
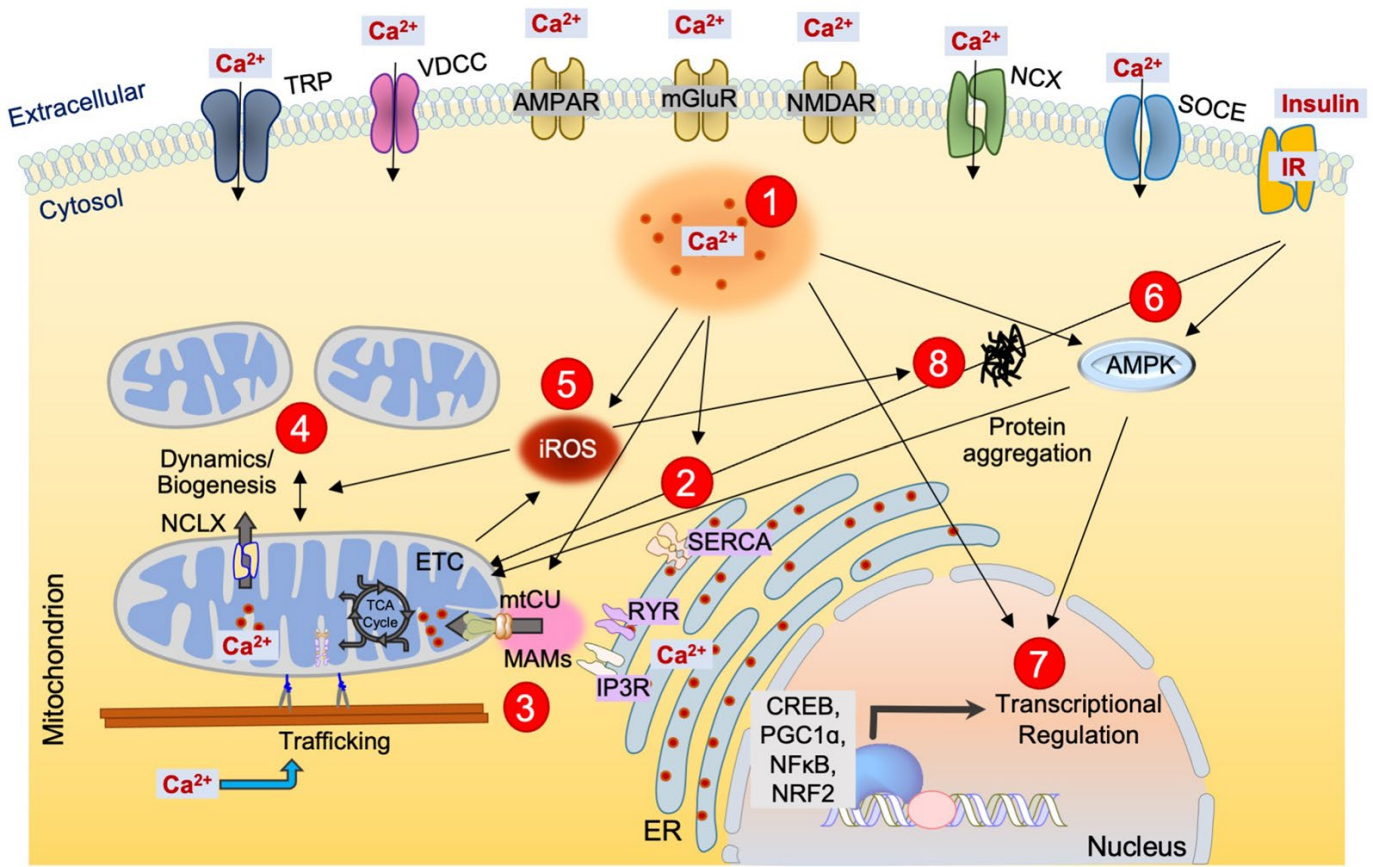
Calcium-centric view of impaired mitochondrial metabolism in NDDs. (1–2) An increase in intracellular calcium by different Ca^2+^ transport systems in the plasma membrane and the endoplasmic reticulum promotes its entry into the mitochondrial matrix via the mtCU. (3) _m_Ca^2+^ enhances the activity of key TCA enzymes, leading to elevated OxPhos and ATP generation. On the other side, insufficient or excessive _m_Ca^2+^ content can impair mitochondrial metabolism in NDDs. The ER plays a crucial role in regulating cellular energetics via the regulated release of Ca^2+^ near sites of ER-mitochondrial contact to support ATP production. (4) The changes in mitochondrial dynamics alter respiratory complex assembly and affect the coupling between respiration and ATP synthesis. (5–8) The production of ROS and activation of AMPK signaling by Ca^2+^ and insulin signaling also constitute the diverse array of signaling pathways that elicit transcription regulation of energy metabolism genes

Above we outlined experimental evidence linking impaired energy metabolism to the initiation or progression of NDDs. This has led to the hypothesis that defects in mitochondrial energy production initiate a cascade of events that causes the neuronal cell death observed in NDDs [173]. However, additional work has raised the possibility that primary defects in other cellular processes may secondarily impair mitochondrial bioenergetics and contribute to NDD pathogenesis [10, 174]. Potential mechanisms that may alter metabolism in NDDs (Fig. 3), and the significance of such altered metabolism for NDDs etiology, are discussed below.

### Calcium signaling

Calcium signaling is required for neuronal function and regulates a range of processes, including neuronal excitability, neurotransmitter release, mitochondrial metabolism, and cell death. Tight control over _i_Ca^2+^ flux is therefore essential for coordinated activity and neuronal homeostasis. As discussed above, altered Ca^2+^ homeostasis has been reported in NDDs and may contribute to neuronal dysfunction and death (reviewed in [175]). This section discusses the impact of altered Ca^2+^ handling in various subcellular compartments and its impact on metabolism.

#### Intracellular calcium

Perturbation of global _i_Ca^2+^ homeostasis alters Ca^2+^ content in compartments, including the ER and mitochondria. Both organelles are implicated in the pathophysiology of NDDs, thus altered _i_Ca^2+^ levels may contribute to NDD progression. Indeed, _i_Ca^2+^ overload is a widely accepted feature of NDDs and is a likely cause of dysfunction and death of the neuronal populations affected by these diseases [176].

Elevated _i_Ca^2+^ content is a common feature of AD and is especially pronounced in neurons containing NFTs [177]. Elevated _i_Ca^2+^ in AD can exert detrimental effects by altering Ca^2+^-dependent signaling. Two examples of Ca^2+^-dependent proteins in neurons are the phosphatase calcineurin and Ca^2+^/calmodulin-dependent protein kinase II (CaMKII). Altered calcineurin and CaMKII signaling have been linked to memory impairment, synaptic loss, and neurodegeneration, all features of AD progression [178]. Such findings have inspired the “Ca^2+^ hypothesis of AD,” which proposes that cellular Ca^2+^ dysregulation is a central driver of disease progression [179, 180].

While Ca^2+^ dysregulation likely precedes neurodegeneration, several reports describe mechanisms by which Aβ directly elevates _i_Ca^2+^ content, suggesting a vicious positive feedback loop that reinforces Ca^2+^ overload. First, Aβ can promote ROS production and subsequent oxidation of membrane lipids that can disrupt cellular ion transport [181]. Second, Aβ peptides may form Ca^2+^-permeable pores in the plasma membrane, allowing for direct influx of Ca^2+^ into the neuron [182]. This idea is supported by the observation that neurites with more Aβ have greater levels of _i_Ca^2+^ [183]. Aβ is also proposed to stimulate Ca^2+^ uptake through L-type voltage-gated Ca^2+^ channels [184], but this notion is still debated [185]. Finally, Aβ may hyperactivate the NMDA receptor, leading to cellular Ca^2+^ overload [186]. Dysregulated Ca^2+^ handling is also implicated in the pathophysiology of PD [187]. Neurons with α-synuclein mutations have increased plasma membrane ion permeability, possibly due to the formation of pores by mutant α-synuclein [188]. Pharmacologic inhibition of Cav1.3 L-type Ca^2+^ channels is protective in animal models of PD [189], suggesting that increased ion channel activity contributes to excess _i_Ca^2+^ entry. Store-operated calcium entry is also impaired in PD and leads to the depletion of ER Ca^2+^ content [175]. Likewise, neuronal Ca^2+^ dysregulation is a common feature of HD [190]. mHtt can stimulate NMDA receptors in medium spiny striatal neurons, potentially leading to excess _i_Ca^2+^ [176]. Also, mHtt binds to and potentiates IP_3_ receptor signaling, enhancing Ca^2+^ release from the ER [191]. These combined effects all tend to deplete the ER of Ca^2+^ and can ultimately enhance store-operated Ca^2+^ entry [192], setting up a continuous cycle that promotes increased _i_Ca^2+^ load.

#### ER calcium

Alterations in _i_Ca^2^ handling in NDDs can cause secondary changes in ER Ca^2+^ load. The ER plays a critical role in regulating cellular energetics via the regulated release of Ca^2+^ near sites of ER-mitochondrial contact. In brief, these discrete sites of ER-mitochondrial apposition (examined further below under “MAMs” or mitochondrial associated membranes) create a microdomain where Ca^2+^ concentration can rise to levels as much as 20 × greater than in the bulk cytosol [193, 194]. This localized, high Ca^2+^ concentration is required for the activation of the _m_Ca^2+^ uptake machinery (gating of the mtCU) and efficient ER-to-mitochondria Ca^2+^ transfer [195]. Inter-organelle Ca^2+^ transport is especially important in regulating _i_Ca^2+^ homeostasis in neurons and is implicated not only in energetic homeostasis but also vesicle trafficking and neurotransmitter release [196, 197]. Thus, any structural disruption in ER-mitochondrial contact sites in NDDs and subsequent perturbation in ER-mitochondrial Ca^2+^ transfer has the potential to exacerbate _i_Ca^2+^ stress and accelerate disease progression. Moreover, altered ER Ca^2+^ content and ER Ca^2+^ release will affect _m_Ca^2+^ content. As discussed in the next section, either insufficient or excessive _m_Ca^2+^ content can impair mitochondrial metabolism and signaling, thus underlying the significance of altered ER Ca^2+^ handling for cellular bioenergetics in NDDs.

#### Mitochondrial calcium

Given the central role of _m_Ca^2+^ in regulating cellular metabolism and survival, it is not surprising that altered _m_Ca^2+^ handling is reported in cellular NDD models. NDDs are universally associated with _m_Ca^2+^ overload, which can impair cellular metabolism by inducing oxidative stress, which itself can impair OxPhos; and by inducing mPTP, which compromises ATP production by collapsing Δψ_m_ [198, 199].

In AD, _m_Ca^2+^ overload can result from excessive ER-to-mitochondrial Ca^2+^ transfer induced by Aβ oligomers [200]. There are also reports that Aβ accumulates in mitochondria and interacts with the matrix mPTP regulator cyclophilin D, thus increasing permeability transition [201, 202] and impairing mitochondrial energetics in a Ca^2+^-independent manner. More recent results from our laboratory indicate that _m_Ca^2+^ efflux is compromised in AD, due to downregulation of NCLX, which further promotes _m_Ca^2+^ overload [77].

Signs of _m_Ca^2+^ overload are observed in cellular models of PD induced by expression of mutant α-synuclein. These include loss of Δψ_m_, cristae structure, and ATP content, features that are exacerbated by simultaneous expression of mutant PINK1 and rescued by pharmacologic blockade of _m_Ca^2+^ uptake [203]. α-synuclein can accumulate within mitochondria and increases _m_Ca^2+^ content, leading to increased ROS production [204]. However, conflicting reports [205] suggest that the effects of α-synuclein on _m_Ca^2+^ homeostasis may be more nuanced. In some cases, α-synuclein may be beneficial by promoting ER-mitochondrial contacts to enhance ER-to-mitochondrial Ca^2+^ transfer and support mitochondrial bioenergetics [206]. Altered _m_Ca^2+^ handling has been suggested in HD, but existing reports have yielded disparate conclusions on this point. The reader is referred to a recent review by Cali et al. [198] for a more detailed discussion.

### Mitochondrial-associated membranes

Mitochondrial-associated membranes (MAMs) are regions where the ER is in close proximation with the outer mitochondrial membrane to allow crosstalk between these organelles. MAMs are particularly important for the exchange of Ca^2+^ and phospholipids, both of which impact ER/mitochondrial function and thus have profound effects on cellular metabolism and overall homeostasis (reviewed in [207]). MAMs are required for the synthesis of lipids such as phosphatidylcholine [208], with the mitochondrion serving as the site of phosphatidylethanolamine (PE) generation, an intermediate in phosphatidylcholine production. In turn, PE is crucial for overall mitochondrial morphology and function [209]. MAMs are also enriched for proteins involved in mitochondrial fission and fusion [210, 211], and so can influence mitochondrial dynamics, morphology, and biogenesis. Likewise, MAMs are important sites for the regulation of mitophagy and the clearance of defective mitochondria [212].

MAMs are often found at synapses, where they may modulate synaptic activity [213]. Efficient ER-to-mitochondria Ca^2+^ transfer is necessary for ATP production and may be especially important for meeting the high energetic demands of synaptic transmission [214] and/or serve as an important mechanism to buffer synaptic Ca^2+^. The ER and mitochondrial membranes are held in apposition at MAMs via a network of tether proteins [215, 216], some of which have been implicated in NDDs.

ER-mitochondrial tethers include the ER-mitochondria encounter structure (ERMES), which was identified in yeast [217]. Mammalian counterparts to the ERMES complex are still being validated, but may include the IP_3_ receptor, phosphofurin acidic cluster sorting protein-2 (PACS-2), B-cell receptor associated protein 31 (Bap31), PDZD8 in the ER, the mitochondrial fission protein Fis1, and the outer mitochondrial membrane protein VDAC [218]. PDZD8 is required for ER-mitochondria tethering, and loss of PDZD8 is sufficient to impact ER-mitochondrial Ca^2+^ dynamics in mammalian neurons [219]. Mitofusin 2 has also been proposed as a MAM tether [220], but this idea remains controversial [221, 222]. Additional proposed tethers include the oxysterol binding-related proteins ORP5 and ORP8, which can interact with mitochondrial protein tyrosine phosphatase interacting protein 51 (PTPIP51) [223]. The OMM protein synaptojanin 2 binding protein (SYNJ2BP) and the ER protein ribosome-binding protein 1 (RRBP1) are proposed to mediate specific interactions between the rough ER and mitochondria [224]. Finally, a tethering complex that may have particular importance in NDDs is comprised of the ER vesicle-associated membrane proteins-associated protein B (VAPB) and mitochondrial PTPIP51 [225, 226].

Altered ER-mitochondrial contacts in NDDs may contribute to disease pathology [227, 228]. Loss of MAM tethers can disrupt ER-mitochondrial Ca^2+^ transfer and so impair mitochondrial metabolism, leading to cellular energy depletion and the activation of autophagy [229, 230]. MAM disruption in NDDs could also lead to energetic compromise by impairing the synthesis of phospholipids important for mitochondrial membranes, such as cardiolipin [208, 231, 232]. This species is enriched in the mitochondrial inner membrane and is critical for proper ETC and ATP synthase function [233–236]. Finally, ER-mitochondrial associations regulate a number of processes that are commonly disrupted in NDDs such as Ca^2+^ handling, inflammation, axonal transport, and mitochondrial function [237]. These observations support the hypothesis that altered ER-mitochondrial communication is a common mechanism underlying NDDs.

The AD-related proteins APP and γ-secretase are all enriched at MAMs [238]. Observations of altered lipid metabolism and Ca^2+^ handling in both FAD and SAD suggest that these proteins may be associated with MAM dysfunction [228, 239]. Altered _i_Ca^2+^ handling in AD could result from enhanced ER-mitochondrial Ca^2+^ transfer. The finding that ER Ca^2+^ concentration is increased in AD supports this view. Finally, altered lipid homeostasis resulting from dysfunctional ER/mitochondrial tethering may also impair mitochondrial energetics in AD. The MAMs of AD brain tissue and cells exhibit increased sphingomyelin hydrolysis by sphingomyelinase, which leads to increased ceramide content [240]. Increased ceramide content in AD appears sufficient to impair mitochondrial respiration [241, 242], as pharmacologic reduction of ceramide levels in AD models can rescue mitochondrial respiration [240]. Specific mechanisms by which elevated ceramide content in mitochondrial membranes may impair respiratory function and cellular bioenergetics have been detailed elsewhere [173].

Furthermore, altered ER-mitochondrial contacts and signaling are reported in PD, leading some to propose that disrupted MAMs are a significant contributor to PD pathogenesis [228, 237]. Proteins that are implicated in familial PD such as α-synuclein, PINK1, and Parkin all alter ER-mitochondrial signaling [243–245]. However, the specific consequences of these alterations on PD pathology are still the subject of active investigation [207].

The protein α-synuclein localizes to MAMs [245] and is thought to influence Ca^2+^ signaling [205, 206] and lipid metabolism [246], ultimately leading to defective ER and mitochondrial function [206]. Whereas wild-type α-synuclein promotes ER-mitochondrial contacts [206], the association of familial PD mutant α-synuclein with MAMs is disrupted. This change may represent one mechanism for compromised MAM structure and function in PD [246]. However, conflicting data suggest that overexpression of either wild-type or mutant α-synuclein can disrupt ER-mitochondrial contacts by binding to VAPB on the ER membrane and interfering with VAPB-PTPIP51 interactions [245]. Disruption of this tether complex can impair mitochondrial energetics because it compromises Ca^2+^ exchange between the two organelles [245]. Similar mechanisms may explain how DJ-1 mutations contribute to early-onset PD [247]. DJ-1 is normally localized to MAMs where it promotes ER-mitochondrial association and facilitates _m_Ca^2+^ uptake [248]. Mutant DJ-1, as seen in PD, may disrupt MAM structure, ER-mitochondrial contacts, _m_Ca^2+^ uptake, and mitochondrial bioenergetics [249]. In addition, mutations in Parkin and PINK1 may initiate PD pathogenesis via effects at MAMs. PINK and Parkin are recruited to sites of contact between ER and defective mitochondria to coordinate their autophagic clearance [244, 250]. Thus, defective PINK or Parkin may disrupt mitochondrial quality control mechanisms that rely on MAM interactions. Over time, this could impair cellular metabolism and contribute to PD pathology due to the accumulation of dysfunctional mitochondria.

### Mitochondrial structural defects

Mitochondrial structure is determined by a precise balance between mitochondrial fusion and fission and membrane dynamics that are mediated by several proteins including mitofusin 1 (MFN1), mitofusin 2 (MFN2), optic atrophy 1 (OPA1), dynamin-related protein 1 (DRP1), mitochondrial fission factor (MFF), and fission 1 protein [251]. During fasting or starvation mitochondria tend to fuse [252] due to inhibition of Drp1 by PKA and AMPK [253, 254]. These changes in mitochondrial structure alter respiratory complex assembly and affect the coupling between respiration and ATP synthesis [252], thereby increasing ATP production efficiency when fuel is scarce.

Defective mitochondrial fission and fusion have been implicated in NDDs [251], and abnormal mitochondrial structure and morphology are reported in AD, PD, and HD [255]. Increased mitochondrial fragmentation is often observed in these conditions. At first consideration, this finding might indicate that neurons in NDDs are well-supplied with metabolic fuels and are fully capable of breaking them down to meet cellular demands for ATP. However, increased mitochondrial fragmentation may instead reflect or even contribute to metabolic dysfunction in NDDs. Cells adapt to prolonged starvation or chronic defects in metabolism with increased mitophagy, which requires mitochondrial fragmentation [256]. Therefore, excess mitochondrial fragmentation may reflect increased stimuli for mitophagy in NDDs (i.e., impaired fuel utilization and/or mitochondrial dysfunction). This is perhaps coupled with impairments in the mitophagic machinery and the consequent accumulation of fragmented organelles. According to the model in which mitochondrial fusion enhances ATP production, a shift in mitochondrial dynamics that favors fission could limit mitochondrial bioenergetics and exacerbate metabolic stress in NDDs.

Several mechanisms are proposed to explain the accumulation of fragmented mitochondria in NDDs. In AD, some reports indicate that net mtDNA content and ETC protein expression are increased [257, 258], suggestive of a net increase in cellular mitochondrial content. This could occur with an increase in mitochondrial biogenesis and/or a decrease in clearance of defective, fragmented mitochondria. For example, APP mutant transgenic mice show upregulation of ETC genes, and Aβ has been shown to increase cellular mtDNA content [258, 259]. On the other hand, other studies report reduced mtDNA content and ETC gene expression in AD brains [260–262]. These disagreements likely reflect differences in the stage of disease examined in these reports. We observed a slight, but non-significant age-dependent decrease in mitochondrial content in AD-mice compared to control mice [77]. Finally, experiments in animal models of AD reveal increased S-nitrosylation of Drp1, which causes hyperactivation of Drp1 and excessive mitochondrial fragmentation [263]. Similar effects of hyperactivated Drp1 have been found in postmortem brain samples from AD patients [263].

The accumulation of fragmented mitochondria in PD could result either from primary mutations in PD-associated genes such as PINK and Parkin [264] or from the pathogenic milieu associated with disease progression. PINK and Parkin cooperate to identify defective mitochondria and target them for degradation via mitophagy [265]. Therefore, impaired clearance and eventual accumulation of dysfunctional mitochondria may be a primary consequence of PD mutations. PINK1 also controls structural plasticity of mitochondrial crista junctions via phosphorylation of the inner mitochondrial membrane protein MIC60/mitofilin [266]. Mutation in PINK1 could impact the PINK1-Mic60 interaction and prevent the recruitment of Parkin to damaged mitochondria in PD. Further, excessive reactive nitrogen species (RNS) production in PD may contribute to the accumulation of fragmented mitochondria by modifying the activity of proteins involved in mitochondrial fission/fusion and mitophagy. For example, S-nitrosylation of Parkin decreases it E3 ubiquitin ligase activity [267], leading to stabilization of its target, Drp1, which promotes mitochondrial fission [268]. Similarly, S-nitrosylation of PINK1 can impair mitophagy [269] and thereby allow fragmented mitochondria to accumulate.

The mechanisms behind altered mitochondrial structure in HD have received less attention. Studies in a transgenic mouse model expressing mutant human HTT suggest a direct transcriptional repression of PGC1α, which could impair mitochondrial biogenesis [270]. Like in AD, increased S-nitrosylation and activation of Drp1 is observed in mouse models of HD [271], and causes excessive mitochondrial fragmentation similar to that seen in HD brains [272]. Recent work suggests that mutant HTT impairs mitophagy in neurons [273], which would also favor the accumulation of dysfunctional mitochondria in HD.

### Oxidative stress

Impaired metabolism in NDDs is linked to the production of RNS and ROS. Multiple hallmarks of NDDs including mitochondrial dysfunction, misfolded proteins, and inflammation are known consequences of elevated RNS/ROS production [274]. The relationship between mitochondrial dysfunction, aberrant ROS signaling, and neurodegeneration has been reviewed elsewhere [275]. Elevated RNS production in NDDs is thought to occur as a result of elevated _i_Ca^2+^ concentration, which increases nitric oxide (NO) production by neuronal nitric oxide synthase (nNOS) and endothelial nitric oxide synthase (eNOS). Excess NO in turn promotes mitochondrial dysfunction, which can exacerbate bioenergetic compromise and accelerate neurodegeneration [276]. This may occur through reversible S-nitrosylation of cysteine residues on proteins important for mitochondrial homeostasis such as Parkin and Drp1, as well as proteins such as Protein disulfide isomerase (PDI) that help to ensure proper protein folding (reviewed in [276]). Nitric oxide can also react with superoxide to form peroxynitrite, which irreversibly modifies tyrosine residues via tyrosine nitration [277].

Nitric oxide inhibits numerous proteins involved in metabolism, providing a mechanistic link between elevated _i_Ca^2+^ levels and altered metabolism in NDDs. NO attenuates glycolysis and fatty acid oxidation via inhibitory S-nitrosylation of key enzymes in these pathways such as GAPDH [274, 276]. Such effects would impede metabolism by limiting carbon input into the TCA cycle. Furthermore, S-nitrosylation of the TCA cycle enzymes citrate synthase, aconitase, isocitrate dehydrogenase, alpha-ketoglutarate dehydrogenase, succinyl-CoA synthetase, succinate dehydrogenase, and malate dehydrogenase has been observed [278, 279] and is often inhibitory [280, 281]. In particular, isocitrate dehydrogenase is a rate-limiting step within the TCA cycle [282], and inhibitory S-nitrosylation of this enzyme could limit TCA cycle flux and overall mitochondrial metabolism. Downstream of the TCA cycle, S-nitrosylation can inhibit ETC complexes I [283–285], IV [286], and V (ATP synthase) [287]. Tyrosine nitration also inhibits all ETC complexes [288, 289]. Thus, excessive RNS production in NDDs can impair mitochondrial metabolism by direct action on multiple targets and pathways.

Much remains to be determined regarding the specific role of mitochondrial RNS stress in the progression of NDDs. Some recent studies support a link between increased NO production and altered mitochondrial activity. Induced pluripotent stem cells expressing the A53T mutation in α-synuclein, which causes familial PD, exhibit decreased mitochondrial respiration that is attributed to aberrant S-nitrosylation of the transcription factor MEF2C, which leads to impaired PGC1α expression [290]. A similar effect of abnormal MEF2 S-nitrosylation is associated with neurodegeneration in AD [291]. Any initial impairment of mitochondrial respiratory activity can trigger excess ROS and RNS production, leading to further oxidative or nitrosative stress [112, 292, 293] that feeds back to impair mitochondrial metabolism. Fitting with this notion, increased ROS production by the ETC is indeed observed in neurodegeneration [274, 294]. While increased ROS production in NDDs may be a direct consequence of increased _m_Ca^2+^ concentration, it is tempting to speculate that increased cellular NO production may also contribute to this effect by initiating ETC dysfunction.

Finally, it is worth noting that data also exist supporting a neuro-protective role for nitric oxide in some NDDs. As reviewed by Calabrese et al., within the context of normal physiology, NO can exert neuro-protective effects via several mechanisms including stimulation of pro-survival Akt and cyclic-AMP-responsive-element binding protein (CREB) signaling pathways, S-nitrosylation of the NMDA receptor to limit cellular Ca^2+^ uptake and excitotoxicity, inhibitory S-nitrosylation of caspases, and the upregulation of heme oxygenase 1 to stimulate cellular antioxidant production [295].

### Transcriptional regulation

Several observations indicate that changes in transcriptional programs contribute to altered metabolism in NDDs. The expression of key energy and metabolism genes, such as components of the ETC, are reduced at both the mRNA and protein level in autopsied AD brains [262]. Furthermore, transcriptional repression of PGC-1α, a transcription coactivator with a central role in mitochondrial biogenesis, is observed in mouse models of HD [270]. These examples illustrate a general phenomenon, common to NDDs, of decreased transcription of genes involved in mitochondrial and oxidative metabolism [296]. Much work remains to determine the mechanisms responsible, but some evidence supports the notion that restoration of transcription is beneficial in NDDs. Specifically, activation of transcription factors including CREB, NF-κB, and NRF2 are protective in murine models of these diseases (reviewed in [174, 297]). It is interesting to note that exercise and aerobic activity can activate some of these neuroprotective transcription factors [174]. Thus, an interesting question is whether impaired locomotion and reduced physical activity in some NDDs diminish the activation of beneficial transcriptional programs, and so drive further transcriptional and metabolic defects.

One example of how metabolic gene transcription may become disrupted in NDDs is by impairment of Peroxisome proliferator-activated receptor (PPAR)-γ co-activator 1α (PGC-1α). As reviewed elsewhere [298], PGC-1α is activated by AMPK during times of metabolic stress, and in concert with the transcription factor NRF-1 increases the expression of nuclear genes involved in mitochondrial biogenesis [299, 300]. PGC-1α also upregulates mitophagic genes [301, 302] and thus can impact mitochondrial quality control, turnover, and net mitochondrial content. NDDs are generally associated with reduced expression of PGC-1α, which likely represents a common mechanism for metabolic impairment in these diseases.

Reduced expression of PGC-1α is observed in Alzheimer’s patients and in the TG2576 mouse model of AD (transgenic expression of the APP Swedish mutation) [303]. Mutant forms of presenilin associated with familial AD are associated with reduced PGC-1α expression [304], while in vitro restoration of PGC-1α in AD cell lines improves overall function [303, 305]. This suggests that diminished PGC-1α function, and perhaps subsequent mitochondrial impairment, contributes to AD pathogenesis. Similar evidence for reduced PGC-1α activity is reported in Parkinson’s disease. PD patients exhibit reduced expression of PGC-1α target genes, such as components of the ETC [306]. In cell and animal models, loss of PGC-1α increases susceptibility to PD [307, 308], while overexpression of PGC-1α protects against neuronal death [306, 309]. Recent work indicates that the protein PARIS (*ZFN746* gene), which is normally ubiquitinated by Parkin, can repress PGC-1α expression [310]. Thus, loss of Parkin in PD may elicit the accumulation of PARIS and downregulation of PGC-1α. In support of this notion, stereotactic injection of recombinant PARIS into the substantia nigra of mice causes neuronal death, but this is prevented by simultaneous injection of exogenous recombinant PGC-1α [310]. Together, these data support the idea that downregulation of PGC-1α is secondary to causative NDD gene mutations, but reduces mitochondria content and disrupts quality control, thereby furthering neuronal dysfunction and disease progression.

Huntington’s disease is more closely linked to defects in PGC-1α signaling than other NDDs. Deletion of PGC-1α in mice causes neurodegeneration and recapitulates symptoms of HD [311, 312], and induction of PGC-1α can rescue HD symptoms in mice[313]. Predictably, HD patients and mouse models display reduced PGC-1α expression and reduced expression of mitochondrial genes [314]. These features can be explained by binding of mutant huntingtin protein to the PGC-1α promotor, which represses PGC-1α transcription [270]. Deletion of PGC-1α in HD mouse models exacerbates neurodegeneration, whereas striatal overexpression of PGC-1α is sufficient to protect against neuronal atrophy [270]. Overall, PGC-1α likely plays a central role in the progression of NDDs, and so is an attractive therapeutic target.

### Insulin signaling

Multiple studies support an association between altered insulin signaling and NDDs. Altered glucose metabolism is common in both AD and PD [174, 315], and both of these diseases are linked to type 2 diabetes [316–318]. Indeed, many of the same risk factors for developing obesity or diabetes (lack of physical activity, excess calorie consumption, etc.) predispose to the development of NDDs, especially AD and PD [319]. Variants in insulin signaling pathway genes, such as AKT [320] and GSK3β [321], increase the risk for PD. Thus, it is possible that diminished insulin responsiveness and impaired glucose utilization contribute to impaired neuronal metabolism in some NDD patients. This represents further evidence that a decline in metabolic health may initiate NDD development.

The glucose transporters GLUT1 (insulin-insensitive) and GLUT3 (insulin-sensitive) are decreased in AD brains [322, 323]. These changes may limit brain glucose uptake and contribute to cognitive impairments in AD [324]. A report that reducing GLUT1 expression in AD mouse models worsens amyloid burden, neurodegeneration, and cognitive function [325] supports this idea. Additionally, insulin deficiency favors phosphorylation of tau and the development of neurofibrillary pathology [326], reinforcing the notion that disrupted insulin signaling promotes AD progression.

In agreement, impaired glucose metabolism is a well-documented feature of PD brains [174], and lower levels of pyruvate oxidation are observed in PD fibroblasts [111]. These effects are recapitulated in animal models of PD [327–329] and may reflect impaired insulin signaling. Activation of AKT, a classical downstream target of insulin signaling, is reduced in the substantia nigra of PD brains and in in vitro cellular models of PD [330–333]. Genetic mutations in proteins linked to PD, including DJ-1 and PINK1, are also associated with diminished AKT signaling [334] and provide further evidence for altered insulin responsiveness in this disease. To the extent that altered insulin/AKT signaling limits carbon (i.e., glucose) metabolism within neurons, it would limit fuel input to the TCA cycle and decrease mitochondrial ATP production [335]. Limited mitochondrial energetics may be just one consequence of diminished glucose uptake or utilization in PD. Dopaminergic neurons do not tolerate glucose starvation [336], and glucose deprivation in vitro is sufficient to cause α-synuclein aggregation and death of dopaminergic neurons [337]. These data support the idea that impaired glucose utilization is an early driver of PD pathology, and may lead not only to impaired mitochondrial metabolism, but also to amyloidosis and neuronal death.

Altered glucose metabolism is an early feature of HD, even though the expression of glucose transporters is normal in initial stages of the disease [153, 338, 339]. This defect is explained by diminished localization of the glucose transporters at the neuronal plasma membrane [340]. Interestingly, defects in metabolism are observed prior to striatal atrophy, and reduced glucose metabolism strongly correlates with HD progression [341–343]. The finding that increasing expression of GLUT3 or enzymes involved in glucose metabolism can protect against the progression of HD [344, 345] strengthens this view.

### AMPK

AMP-activated protein kinase (AMPK) is a master cellular energy sensor and has a critical role in maintaining metabolic homeostasis. AMPK is activated in response to changes indicative of energetic stress (e.g. increased AMP/ATP ratio, hypoxia, a drop in cellular pH, increased _i_Ca^2+^ concentration, etc.) and via phosphorylation by the kinases LKB1, CaMKKβ, and TAK-1 (reviewed in [346, 347]). AMPK exerts multiple effects to stimulate ATP production, such as stimulating glucose uptake, glycolysis, and glucose and fatty acid oxidation, while at the same time limiting cellular ATP consumption by inhibiting fatty acid and cholesterol production [298, 347]. AMPK also promotes long-term increases in mitochondrial energy production by phosphorylating PGC-1α and the fork-head box O (FOXO) transcription factor to stimulate mitochondrial biogenesis [299, 300, 309, 348–351].

AMPK is activated by ROS, which as previously detailed are elevated in many NDDs [352, 353]. Since AMPK activation can exacerbate ROS production, this may set up a positive feedback loop leading to further oxidative stress and metabolic impairment [354]. Thus, AMPK has the potential to exert both positive and detrimental effects in NDDs. Data supporting both positive and negative aspects of AMPK activation exist for most NDDs, and the net positive versus detrimental outcomes of AMPK activation likely varies between different disorders.

Elevated AMPK activity has been reported in the brains of the APPswe/PS1dE9 and APPswe,ind^,^ mouse models of AD [355, 356]. Several mechanisms have been proposed to explain how this occurs. First, any existing mitochondrial dysfunction due to Aβ accumulation [201, 357, 358], or decreased mitochondrial biogenesis and increased fragmentation [359], could cause energetic stress and AMPK activation. Second, Aβ causes excessive _i_Ca^2+^ flux due to activation of the NMDA receptor, which can activate the AMPK-kinase, CaMKKβ [355, 360]. Third, elevated ROS production [201] and elevated _i_Ca^2+^ [361] downstream of mitochondrial dysfunction can increase AMPK activity in AD. Finally, increased NADPH oxidase activity is observed in AD brains and is proposed to activate AMPK [362].

Although AMPK activation may initially be an adaptive response to alleviate energetic stress in AD, most data indicate that abnormal AMPK activation eventually turns detrimental. For example, AMPK can increase Aβ expression, and Aβ can further activate AMPK, which can suppress long-term potentiation and impair memory [298]. Similarly, AMPK activation increases the phosphorylation of tau [363] and reduces the binding of tau to microtubules [360, 363], potentially accelerating tauopathy. These effects help explain why pharmacologic inhibition of AMPK with compound C or genetic ablation of AMPKα2 subunits is beneficial in the APPswe/PS1dE9 mouse model of AD [364]. Further data in support of a detrimental role of AMPK in AD comes from studies showing that treatment of AD mice with the AMPK activator metformin results in transcriptional upregulation of β-secretase, leading to increased Aβ formation and worsened memory [365, 366]. These studies suggest that AMPK activity furthers metabolic impairment and AD progression by contributing to, or propagating, the pathogenic milieu. It is worth noting that some beneficial effects of AMPK activation have also been observed in AD models. In *Drosophila*, Aβ suppresses AMPK signaling [367], suggesting that insufficient rather than excessive AMPK activity may contribute to AD progression. Consistent with this notion, activation of AMPK by AICAR in rat cortical neurons decreases Aβ content, and knockout of the AMPKα2 subunit increases Aβ production [368]. AMPK activation in response to leptin signaling reduces tau phosphorylation [369, 370], and compounds that activate AMPK, such as resveratrol and metformin, stimulate Aβ metabolism, reduce mitochondrial dysfunction, and improve AD pathology [371–373]. The conflicting data regarding the beneficial versus detrimental roles of AMPK in AD may reflect disparities among the various models and cell types studied with respect to differential expression of AMPK subunit isoforms and their regulation, relative activity, and specific cellular targets, or may be due to temporal differences in disease progression.

AMPK likely also has divergent effects on energetics and neurodegeneration in PD depending on the model or stage of the disease [374]. AMPK is activated in mice treated with MPP^+^, a common in vivo model for PD, as well as in SH-SY5Y cells (human neuroblastoma cell line) treated with MPP^+^ in vitro [375]. The available data suggest that AMPK activation is beneficial and promotes cell survival [375, 376]. For example, pharmacologic inhibition of AMPK increases neuronal cell death in response to MPP^+^ treatment, whereas AMPK overexpression promotes cell survival [375]. In line with these findings, AMPK cooperates with Parkin to maintain mitochondrial quality control and promote neuronal survival [374]. However, the possibility of detrimental effects of AMPK activation to cellular energetics and survival in PD cannot be fully excluded. For instance, AMPK activation in response to cellular ATP depletion is implicated in the degeneration of dopaminergic neurons [377]. Thus, more work is needed to elucidate the precise role of AMPK activation in PD and clarify whether it promotes or impairs metabolic function and overall cellular viability.

The brains of HD patients and HD mouse models exhibit excessive AMPK activation [354, 378, 379]. Both mitochondrial dysfunction and oxidative stress are reported in HD [380], and these defects may both contribute to AMPK activation, or vice-versa be consequences of excessive AMPK activity. mHtt protein likely initiates metabolic stress leading to downstream AMPK activation. mHtt can aggregate on mitochondrial membranes and disrupt _m_Ca^2+^ flux, causing Ca^2+^-dependent oxidative stress [381, 382]. mHtt aggregates also decrease Complex II and Complex III activity [170, 383, 384] and impair mitochondrial trafficking [385]. All these effects can disrupt cellular energy balance and trigger AMPK activation. The existing literature suggests that AMPK activation is detrimental in HD, culminating in neuronal apoptosis [354, 379]. This effect may be related to the suppression of the survival gene Bcl-2 [379]. Whether excess AMPK activity is also toxic due to metabolic perturbations remains to be determined.

### Neuroinflammation

Previous studies have indicated that optimal brain function requires coordinated signaling between neurons and glial cells, and disturbances in paracellular communication can contribute to NDDs development. In addition, the inflammatory hypothesis suggests that the activation of microglia is a driving force for neuroinflammation and mitochondrial dysfunction in NDDs. In turn, mitochondrial dysfunction can promote inflammation (reviewed in [386, 387]).

Microglia are specialized brain macrophages with a primary function in host defense including the removal of cellular debris, metabolic waste, pathogens, and neurotoxins [388]. Microglia are dynamic cells that can change their shape and undergo phenotypic transformation (activation) in response to infection or injury. In the resting homeostatic state, microglia exhibit a ramified structure with branching processes for surveillance of the local environment [389]. After activation, microglia become highly mobile, assuming an amoeboid form with short thickened processes, and phagocytose cell debris, secrete proinflammatory mediators, such as cytokines, and generate ROS to potentiate acute inflammation [389]. While thought to serve a protective role during acute inflammation, persistent microglia activation contributes to chronic neuroinflammation and redox imbalance associated with NDDs, resulting in mitochondrial dysfunction [390, 391]. This elicits a positive feedback loop where mitochondrial-generated superoxide potentiates microglial activation, initiating further ROS production. As previously discussed ROS can promote posttranslational modifications of TCA cycle enzymes and induce mtDNA mutations, which in turn can compromise energetics and trigger mitochondrial dysfunction [392].

Fuel sources are thought to be altered in NDDs, resulting in cell-specific metabolic shifts to maintain ATP production [393]. The minimal experimental data available suggests that similar metabolic pathway switching occurs during microglial activation. Transcriptomic studies suggest that microglia express all the required genes for OxPhos and glycolysis [394]. Limited data suggest that microglia undergo reprogramming during activation to favor glycolysis over OxPhos [395–397]. Lipopolysaccharide (LPS) activation of transformed mouse microglial cells (BV-2 cells) decreased OxPhos and lowered ATP production with a concomitant increase in lactate production [397]. These observations are bolstered by the finding of increased lactate production and glucose uptake (high expression of GLUT1 and GLUT4) in activated microglia, favoring aerobic glycolysis and an increase in pentose phosphate pathway flux [396].

Multiple inflammatory mediators resulting from chronic neuroinflammation can affect mitochondrial energy metabolism and mitochondrial dynamics, thereby contributing to NDDs (reviewed in [387]). However, the direct molecular mechanisms are still not precise in neuronal and glial cells by which these inflammatory factors impact mitochondrial metabolism. Few reports in non-neuronal cells suggest that inflammatory mediators, TNF and IL-1β, reduce the activity of TCA cycle enzymes including PDH and α-KGDH, with a concurrent reduction in Complex I and II activity [398]. α-KGDH activity is reported to be reduced by an inflammation-derived oxidant, myeloperoxidase, that is upregulated in microglia in AD brain tissue [399]. This suggests that inflammatory factors can impact mitochondrial metabolism in glial cells in AD. In addition, TNF has been reported to reduce the expression of PGC-1α in non-neuronal cells [400]. However, the direct interplay between neuroinflammation and mitochondrial metabolism in different NDDs remains poorly understood and thus warrants further investigation.

### Peroxisomal lipid metabolism

Metabolic dysregulation associated with peroxisome dysfunction may contribute to the development of NDDs. Peroxisomes are highly dynamic and important metabolic organelles that can directly communicate with mitochondria and contribute to cellular lipid metabolism, e.g., the oxidation of very-long-chain fatty acids (VLCFAs), synthesis of phospholipids, such as plasmalogen/ether lipids (myelin sheath lipids) and docosahexaenoic acid (DHA), and the regulation of redox and inflammatory signaling. Furthermore, the brain is a lipid-rich organ, and myelin sheaths are rich in plasmalogens/ether lipids synthesized in peroxisomes. Therefore, slight alterations in peroxisomal lipid metabolism may represent significant mechanisms contributing to changes in neuronal function (reviewed in [401]).

In AD, alternations in lipid homeostasis/peroxisome function include significantly decreased levels of plasmalogens and DHA and increased levels of VLCFA. The severity of these alternations correlates with the progression of disease [402, 403] and has been shown to change cell membrane properties and increase intracellular cholesterol levels. These changes increase β-secretase and γ-secretase activities, resulting in enhanced Aβ generation, tau hyperphosphorylation, synaptic dysfunction, and neuroinflammation [404, 405]. In addition, peroxisomal β-oxidation inhibition increased Aβ generation in rat brains (reviewed in [405]). Similarly, severe alterations in lipid composition (reductions in DHA and plasmalogens) of frontal cortex lipid rafts from PD patients have been reported [406]. Reductions in ether lipids decreased Ca^2+^-dependent neurotransmitter release and the respiratory capacity of synaptic mitochondria [407]. Therefore, it is possible that the decrease of ether lipids in mitochondrial membranes might disrupt OxPhos complexes and thus ATP generation sufficiently to compromise neurotransmission. However, overall the role of peroxisomal lipid metabolism in NDDs is poorly described. Further studies are required to determine whether peroxisomal lipid dysfunction directly contributes to disease etiology or is a secondary phenomenon. We refer the reader to another recent review for a detailed overview of the peroxisomal lipid metabolism in NDDs and its metabolic cooperation with mitochondria [405, 408].

## Modulation of mitochondrial function as a possible therapeutic target for neurodegeneration

As discussed earlier, dysregulation in _m_Ca^2+^ homeostasis might be an upstream event causing mitochondrial dysfunction in NDDs. For this reason, various combinations of modulators aimed at targeting or correcting defects in _m_Ca^2+^ exchange or restoring mitochondrial function/energy metabolism may serve as therapies to prevent the development of NDDs. Possible therapeutic strategies, summarized in Table 1, include reducing _m_Ca^2+^ uptake, enhancing _m_Ca^2+^ efflux, and preserving mitochondrial architecture/functions (such as the assembly of respiratory chain complexes and the ATP synthase), bioenergetics, axonal transport of mitochondria, and mitochondrial proteostasis. However, it is still unclear whether increasing _m_Ca^2+^ efflux or reducing mitochondrial _m_Ca^2+^ uptake will be superior for neuroprotection. Both are sufficient to limit _m_Ca^2+^ overload and correct _m_Ca^2+^ dysregulation. Still, a few points need careful consideration, such as if modulators of mitochondrial _m_Ca^2+^ homeostasis will negatively impact Ca^2+^-dependent physiological functions, such as TCA cycle flux and mitochondrial dynamics. It should also be noted that different NDDs might have disease-specific regulation of mtCU channel activity, which requires more detailed experimentation. Beyond this, cellular heterogeneity in mitochondrial function should also be considered; for example, axonal and synaptic mitochondria are reported to be involved in Ca^2+^ buffering and presynaptic transmission, whereas the soma is the primary site for mitochondrial quality control. Therefore, a proper understanding and regulation of the mtCU, or a combination of modulators that aim to increase _m_Ca^2+^ buffer capacity and still maintain energetics may be necessary to maintain efficient synaptic transmission and effectively treat NDDs.

**Table 1:** Potential mitochondrial targets for neurodegenerative therapy

Possible mitochondrial targets		Function	Effect	Possible outcome
mtCU-dependent _m_Ca^2+^ influx	MCU inhibitor	MCU encodes the channel-forming portion of the mtCU complex, and loss of MCU completely ablates all channel function [71, 72]	Reduce _m_Ca^2+^ uptake	Modulation of mtCU dependent _m_Ca^2+^ uptake mechanisms can reduce pathogenic _m_Ca^2+^ overload, maintain mitochondrial Ca^2+^ homeostasis and preserve structure and function
	MCUB activator	MCUB, a paralog of MCU, exerts a dominant-negative effect and negatively regulates the mtCU by replacing MCU subunits [411]		
	MICU1 modulator	MICU1 prominently regulates the Ca^2+^ threshold of the mitochondrial calcium uniporter complex (mtCU) [412, 413] and modulates mitochondrial ultrastructure and cristae organization independent of mtCU [414]		
	MICU3 modulator	MICU3, a brain-specific regulator of _m_Ca^2+^ uptake, act as an activator at low _i_Ca^2+^ levels [415]		
	EMRE inhibitor	EMRE, an essential mtCU regulator and loss of EMRE will reduce overall mtCU formation [416]		
_m_Ca^2+^ efflux	NCLX activator	A mitochondrial Na^+^/Ca^2+^ exchanger acts as a primary route of Ca^2+^ efflux (3-Na^+^ in/1-Ca^2+^ out) (NCLX) [73, 74]	Enhance _m_Ca^2+^ efflux	Enhancing _m_Ca^2+^ efflux can reduce pathogenic _m_Ca^2+^ overload and its associated mitochondrial dysfunction
mPTP opening	Cyclophilin D inhibitor	Inhibits mPTP opening and loss of Δψm	Reduce cell death	Inhibiting mPTP will decrease Ca^2+^-induced mitochondrial swelling, ROS, and cell death
Complex formation (scaffolds)	MICOS stabilizer (Mic10 and Mic60)	MICOS is a mitochondrial contact site and cristae organizing system [417] that are regulated by _m_Ca^2+^ [418]	Preserve mitochondrial function	Maintaining the MICOS function will preserve respiratory chain complexes, the ATP synthase, mitochondrial architecture, biogenesis, bioenergetics, and function
Proteases	m-AAA proteases activator (AFG3L2, paraplegin, YME1L, PARL, HTRA2	m-AAA proteases regulate the assembly of the mitochondrial calcium uniporter complex (mtCU) [419] and OxPhos complex [420]	Mitochondrial protein quality control mechanisms and mitochondrial proteostasis	Activation of m-AAA proteases will maintain mitochondrial membrane dynamics, axonal transport of mitochondria and can stabilize the mitochondrial genome and the synthesis of mitochondrially encoded subunits of the ETC and preserve mitochondrial morphology
MAMs	PDZD8 Inhibitor	An ER protein present at ER-mitochondria contacts and required for Ca^2+^ uptake by mitochondria [219]		Reduced ER-mitochondria Ca^2+^ transfer could protect against _m_Ca^2+^ overload mediated cell death

## Challenges, conclusions, and future research directions

A more detailed and nuanced understanding of the cellular and molecular mechanisms altering neuronal mitochondrial metabolism in NDDs is still needed. Several challenging questions remain to be answered, such as (1) How can mitochondrial defects be central in so many different NDDs with diverse etiologies and pathologies? (2) How does mitochondrial dysfunction contribute to protein aggregation? (3) Do metabolic defects cause neurodegeneration, or does neuronal dysfunction result in metabolic defects? (4) What are the cellular and molecular events that initiate mitochondrial dysfunction in neurodegeneration? (5) How do neurons sense bioenergetic crisis during stress or in pathology? (6) How do cell specific metabolic profiles impact cellular crosstalk in the context of disease progression? 8) What upstream and downstream signaling pathways are involved at the time of bioenergetic crisis in different NDDs? 9) What are the best models to decipher these events for translation into humans?

Here we summarized how numerous cellular events that are compromised during neurodegeneration all require high levels of ATP (e.g., postsynaptic signaling, axonal transport, protein clearance mechanisms and neurotransmission). We also reviewed the evidence that supports the notion that _m_Ca^2+^ and metabolic impairments are primary cellular defects in NDD pathogenesis. Interestingly, all NDDs share common mechanisms of disease pathology and mitochondrial defects may be a central mechanism in NDD progression. However, it remains enigmatic how mitochondrial dysfunction contributes directly to protein aggregation and brain region- and cell type-specific dysfunction in NDDs and whether mitochondrial dysfunction is causal or the consequence of the underlying pathology. Here, we propose a positive feedback loop between mitochondrial defects and disease pathology that explains numerous mechanisms of NDDs. It’s plausible that early mitochondrial dysfunction directly affects protein aggregation through ATP-dependent proteostasis machinery (protein synthesis, folding, and degradation) together with oxidative stress and inflammation and promotes cell-type-specific loss due to mitochondrial death signaling or due to metabolic and energetic dysfunction. Recent reports suggest that aggregation-prone proteins shuttle to mitochondria and mitochondrial protein quality control may alleviate protein aggregation. Mitochondrial dysfunction leading to the failure of mitochondrial proteostasis could be another crucial factor for pathology-specific protein aggregation. For a detailed mechanism by which mitochondrial dysfunction leads to protein aggregation, we refer the reader to other recent reviews [409, 410]. A cell type/brain region-specific regulation of mitochondrial function and _m_Ca^2+^ signaling is lacking and how this contributes to different disease pathologies is entirely unexplored. A complete understanding and precise regulation of mtCU function in different NDDs could eventually help define mechanisms in tissue- and cell-type-specific NDDs.

Another major challenge in NDDs research is selecting experimental models that recapitulate the pathological features of human disease. For decades, animal models have been essential because they are sufficient to recapitulate human genetic mutations and mimic critical clinical features. These model systems have provided access to define in vivo systemic interactions, and study developmental, metabolic, and behavioral outcomes, which is not possible in cellular systems or patients. Arguably, discoveries in animal models have led to a better understanding of the molecular mechanisms of disease pathogenesis but failed to translate in humans. However, the failure to translate insights gained from mouse models into humans is not always due to flaws of the animal model per se. For example, many of these studies lacked detailed causal experimentation and did not exclude other variable factors. Other viable alternatives that can help recapitulate human pathophysiology such as the study of postmortem human brains and human iPSCs, and organoids may help as translational stepping stones to therapy. The postmortem human brain is particularly helpful to quantify cellular and molecular markers of disease and the pathology of neural processes. However, the access of these samples is limited, and the quality of the tissue is impacted by the donor’s condition pre-mortem, postmortem interval, collection time, and maintenance conditions all of which can introduce confounding variables. Human iPSCs are a versatile tool to model human neurons and suitable for human in vitro studies, such as high-throughput drug screening. Still, they cannot enable in vivo cellular physiology which takes into account organ and cellular crosstalk and the complex milieu of the complete organism. We believe an assortment of models, including robust animal models and three-dimensional cellular systems, will help better define the pathogenesis of NDDs and enable more thorough testing of drugs and therapies for clinical translation. Indeed, it is critical to generate robust animal models that phenocopy either the familial or non-familial forms of these disorders. An increase in proper causal experimental design using robust engineered animal models that recapitulate the complexity of an entire nervous system, including a full complement of neuronal circuits, glial complexity, and the vascular and immunologic components, will provide valuable insight into how mitochondrial metabolism impacts disease pathogenesis. In conclusion, a better understanding of metabolic regulation, identification of mitochondrial targets (see Table 1), and determining the precise temporal order of pathological cellular events is of paramount importance to development of novel therapeutic targets to combat NDDs.

## Abbreviations

NDDs: Neurodegenerative diseases
AD: Alzheimer’s disease
PD: Parkinson’s disease
HD: Huntington’s disease
PET: Positron emission tomography
Aβ: Amyloid-beta
NFTs: Neurofibrillary tangles
OxPhos: Oxidative phosphorylation
ETC: Electron transport chain
PDH: Pyruvate dehydrogenase
α-KGDH: Alpha-ketoglutarate dehydrogenase
ICDH: Isocitrate dehydrogenase
SDH: Succinate dehydrogenase
MDH: Malate dehydrogenase
Δψm: Mitochondrial membrane potential
COX: Cytochrome-c-oxidase
_i_Ca^2^^+^: Intracellular calcium
mtCU: Mitochondrial calcium uniporter channel
_m_Ca^2^^+^: Mitochondrial calcium
NCLX: Mitochondrial Na^+^/Ca^2^^+^ exchanger
MCU: Mitochondrial calcium uniporter
mPTP: Mitochondrial permeability transition pore
PMCA: Plasma membrane Ca^2^^+^ ATPase
NCX: Na^+^/Ca^2^^+^ exchanger
TRP: The transient receptor potential
VDCC: Voltage-gated calcium channels
AMPAR: α-Amino-3-hydroxy-5-methyl-4-isoxazolepropionic acid receptor
mGluR: Metabotropic glutamate receptors
NMDAR: N-Methyl-D-aspartate receptor
NCX: Na^+^/Ca^2^^+^ exchanger
SOCE: Store-operated calcium entry
IP_3_R: Inositol 1,4,5-trisphosphate receptor
RYR: Ryanodine receptor
SERCA: Sarco/endoplasmic reticulum Ca^2^^+^-ATPase
TCA: Tricarboxylic acid cycle
MAMs: Mitochondrial-associated membranes
ROS: Reactive oxygen species
RNS: Reactive nitrogen species
AMPK: AMP-activated protein kinase
PGC-1α: Peroxisome proliferator-activated receptor (PPAR)-γ co-activator 1α
mtDNA: Mitochondrial DNA

## References

[1] Kety SS (1957) The general metabolism of the brain in vivo. Metabolism of the nervous system. Elsevier, pp 221–237

[2] Oyarzabal A, Marin-Valencia I (2019). Synaptic energy metabolism and neuronal excitability, in sickness and health. J Inherit Metab Dis.

[3] Vergara RC, Jaramillo-Riveri S, Luarte A, Moenne-Loccoz C, Fuentes R, Couve A, Maldonado PE (2019). The energy homeostasis principle: neuronal energy regulation drives local network dynamics generating behavior. Front Comput Neurosci.

[4] Bordone MP, Salman MM, Titus HE, Amini E, Andersen JV, Chakraborti B, Diuba AV, Dubouskaya TG, Ehrke E, Espindola de Freitas A (2019). The energetic brain—a review from students to students. J Neurochem.

[5] Formentini L, Pereira MP, Sanchez-Cenizo L, Santacatterina F, Lucas JJ, Navarro C, Martinez-Serrano A, Cuezva JM (2014). In vivo inhibition of the mitochondrial H+-ATP synthase in neurons promotes metabolic preconditioning. EMBO J.

[6] Motori E, Atanassov I, Kochan SMV, Folz-Donahue K, Sakthivelu V, Giavalisco P, Toni N, Puyal J, Larsson NG (2020). Neuronal metabolic rewiring promotes resilience to neurodegeneration caused by mitochondrial dysfunction. Sci Adv.

[7] Burmistrova O, Olias-Arjona A, Lapresa R, Jimenez-Blasco D, Eremeeva T, Shishov D, Romanov S, Zakurdaeva K, Almeida A, Fedichev PO (2019). Targeting PFKFB3 alleviates cerebral ischemia-reperfusion injury in mice. Sci Rep.

[8] Herrero-Mendez A, Almeida A, Fernandez E, Maestre C, Moncada S, Bolanos JP (2009). The bioenergetic and antioxidant status of neurons is controlled by continuous degradation of a key glycolytic enzyme by APC/C-Cdh1. Nat Cell Biol.

[9] Jack CR, Bennett DA, Blennow K, Carrillo MC, Dunn B, Haeberlein SB, Holtzman DM, Jagust W, Jessen F, Karlawish J (2018). NIA-AA research framework: toward a biological definition of Alzheimer's disease. Alzheimer's Dementia.

[10] Mattson MP, Pedersen WA, Duan W, Culmsee C, Camandola S (1999). Cellular and molecular mechanisms underlying perturbed energy metabolism and neuronal degeneration in Alzheimer's and Parkinson's diseases. Ann NY Acad Sci.

[11] Spinelli JB, Haigis MC (2018). The multifaceted contributions of mitochondria to cellular metabolism. Nat Cell Biol.

[12] Golpich M, Amini E, Mohamed Z, Azman Ali R, Mohamed Ibrahim N, Ahmadiani A (2017). Mitochondrial dysfunction and biogenesis in neurodegenerative diseases: pathogenesis and treatment. CNS Neurosci Ther.

[13] Rossi A, Rigotto G, Valente G, Giorgio V, Basso E, Filadi R, Pizzo P (2020). Defective mitochondrial pyruvate flux affects cell bioenergetics in Alzheimer's disease-related models. Cell Rep.

[14] Cunnane SC, Trushina E, Morland C, Prigione A, Casadesus G, Andrews ZB, Beal MF, Bergersen LH, Brinton RD, de la Monte S (2020). Brain energy rescue: an emerging therapeutic concept for neurodegenerative disorders of ageing. Nat Rev Drug Discov.

[15] McFarthing K, Buff S, Rafaloff G, Dominey T, Wyse RK, Stott SRW (2020). Parkinson's disease drug therapies in the clinical trial pipeline: 2020. J Parkinsons Dis.

[16] Travessa AM, Rodrigues FB, Mestre TA, Ferreira JJ (2017). Fifteen years of clinical trials in Huntington's disease: a very low clinical drug development success rate. J Huntington's Dis.

[17] Perez MJ, Ponce DP, Aranguiz A, Behrens MI, Quintanilla RA (2018). Mitochondrial permeability transition pore contributes to mitochondrial dysfunction in fibroblasts of patients with sporadic Alzheimer's disease. Redox Biol.

[18] Teo E, Ravi S, Barardo D, Kim HS, Fong S, Cazenave-Gassiot A, Tan TY, Ching J, Kovalik JP, Wenk MR (2019). Metabolic stress is a primary pathogenic event in transgenic Caenorhabditis elegans expressing pan-neuronal human amyloid beta. Elife.

[19] Fao L, Rego AC (2020). Mitochondrial and redox-based therapeutic strategies in Huntington's disease. Antioxid Redox Signal.

[20] Mi Y, Qi G, Brinton RD, Yin F (2020). Mitochondria-targeted therapeutics for Alzheimer's disease: the good, the bad, the potential. Antioxid Redox Signal.

[21] Weissig V (2020). Drug development for the therapy of mitochondrial diseases. Trends Mol Med.

[22] Britti E, Delaspre F, Tamarit J, Ros J (2018). Mitochondrial calcium signalling and neurodegenerative diseases.

[23] Mehta D, Jackson R, Paul G, Shi J, Sabbagh M (2017). Why do trials for Alzheimer's disease drugs keep failing? A discontinued drug perspective for 2010–2015. Expert Opin Investig Drugs.

[24] Panza F, Lozupone M, Watling M, Imbimbo BP (2019). Do BACE inhibitor failures in Alzheimer patients challenge the amyloid hypothesis of the disease?. Expert Rev Neurother.

[25] Selkoe DJ, Hardy J (2016). The amyloid hypothesis of Alzheimer's disease at 25 years. EMBO Mol Med.

[26] Cai Q, Tammineni P (2017). Mitochondrial aspects of synaptic dysfunction in Alzheimer's disease. J Alzheimers Dis.

[27] Liu C, Song X, Nisbet R, Gotz J (2016). Co-immunoprecipitation with tau isoform-specific antibodies reveals distinct protein interactions and highlights a putative role for 2N tau in disease. J Biol Chem.

[28] Manczak M, Reddy PH (2012). Abnormal interaction of VDAC1 with amyloid beta and phosphorylated tau causes mitochondrial dysfunction in Alzheimer's disease. Hum Mol Genet.

[29] Giannakopoulos P, Herrmann FR, Bussiere T, Bouras C, Kovari E, Perl DP, Morrison JH, Gold G, Hof PR (2003). Tangle and neuron numbers, but not amyloid load, predict cognitive status in Alzheimer's disease. Neurology.

[30] Guillozet AL, Weintraub S, Mash DC, Mesulam MM (2003). Neurofibrillary tangles, amyloid, and memory in aging and mild cognitive impairment. Arch Neurol.

[31] Mostafavi S, Gaiteri C, Sullivan SE, White CC, Tasaki S, Xu J, Taga M, Klein HU, Patrick E, Komashko V (2018). A molecular network of the aging human brain provides insights into the pathology and cognitive decline of Alzheimer's disease. Nat Neurosci.

[32] Neff RA, Wang M, Vatansever S, Guo L, Ming C, Wang Q, Wang E, Horgusluoglu-Moloch E, Song WM, Li A (2021). Molecular subtyping of Alzheimer's disease using RNA sequencing data reveals novel mechanisms and targets. Sci Adv.

[33] Cummings J, Lee G, Ritter A, Sabbagh M, Zhong K (2020). Alzheimer's disease drug development pipeline: 2020. Alzheimers Dement (NY).

[34] Jansen IE, Savage JE, Watanabe K, Bryois J, Williams DM, Steinberg S, Sealock J, Karlsson IK, Hagg S, Athanasiu L (2020). Author Correction: Genome-wide meta-analysis identifies new loci and functional pathways influencing Alzheimer's disease risk. Nat Genet.

[35] Swerdlow RH (2020). The mitochondrial hypothesis: dysfunction, bioenergetic defects, and the metabolic link to Alzheimer's disease. Int Rev Neurobiol.

[36] Calkins MJ, Manczak M, Mao P, Shirendeb U, Reddy PH (2011). Impaired mitochondrial biogenesis, defective axonal transport of mitochondria, abnormal mitochondrial dynamics and synaptic degeneration in a mouse model of Alzheimer's disease. Hum Mol Genet.

[37] Fecher C, Trovo L, Muller SA, Snaidero N, Wettmarshausen J, Heink S, Ortiz O, Wagner I, Kuhn R, Hartmann J (2019). Cell-type-specific profiling of brain mitochondria reveals functional and molecular diversity. Nat Neurosci.

[38] Schmukler E, Solomon S, Simonovitch S, Goldshmit Y, Wolfson E, Michaelson DM, Pinkas-Kramarski R (2020). Altered mitochondrial dynamics and function in APOE4-expressing astrocytes. Cell Death Dis.

[39] Perez MJ, Ponce DP, Osorio-Fuentealba C, Behrens MI, Quintanilla RA (2017). Mitochondrial bioenergetics is altered in fibroblasts from patients with sporadic Alzheimer's disease. Front Neurosci.

[40] Manczak M, Kandimalla R, Yin X, Reddy PH (2018). Hippocampal mutant APP and amyloid beta-induced cognitive decline, dendritic spine loss, defective autophagy, mitophagy and mitochondrial abnormalities in a mouse model of Alzheimer's disease. Hum Mol Genet.

[41] Hardy JA, Higgins GA (1992). Alzheimer's disease: the amyloid cascade hypothesis. Science.

[42] de Leon MJ, Ferris SH, George AE, Christman DR, Fowler JS, Gentes C, Reisberg B, Gee B, Emmerich M, Yonekura Y (1983). Positron emission tomographic studies of aging and Alzheimer disease. AJNR Am J Neuroradiol.

[43] Ferris SH, de Leon MJ, Wolf AP, Farkas T, Christman DR, Reisberg B, Fowler JS, Macgregor R, Goldman A, George AE (1980). Positron emission tomography in the study of aging and senile dementia. Neurobiol Aging.

[44] Foster NL, Chase TN, Fedio P, Patronas NJ, Brooks RA, Di Chiro G (1983). Alzheimer's disease: focal cortical changes shown by positron emission tomography. Neurology.

[45] Sorbi S, Bird ED, Blass JP (1983). Decreased pyruvate dehydrogenase complex activity in Huntington and Alzheimer brain. Ann Neurol.

[46] Yao J, Irwin RW, Zhao L, Nilsen J, Hamilton RT, Brinton RD (2009). Mitochondrial bioenergetic deficit precedes Alzheimer's pathology in female mouse model of Alzheimer's disease. Proc Natl Acad Sci USA.

[47] Gibson GE, Starkov A, Blass JP, Ratan RR, Beal MF (2010). Cause and consequence: mitochondrial dysfunction initiates and propagates neuronal dysfunction, neuronal death and behavioral abnormalities in age-associated neurodegenerative diseases. Biochim Biophys Acta.

[48] Fisar Z, Hansikova H, Krizova J, Jirak R, Kitzlerova E, Zverova M, Hroudova J, Wenchich L, Zeman J, Raboch J (2019). Activities of mitochondrial respiratory chain complexes in platelets of patients with Alzheimer's disease and depressive disorder. Mitochondrion.

[49] Kish SJ, Bergeron C, Rajput A, Dozic S, Mastrogiacomo F, Chang LJ, Wilson JM, DiStefano LM, Nobrega JN (1992). Brain cytochrome oxidase in Alzheimer's disease. J Neurochem.

[50] Parker WD, Filley CM, Parks JK (1990). Cytochrome oxidase deficiency in Alzheimer's disease. Neurology.

[51] Bubber P, Haroutunian V, Fisch G, Blass JP, Gibson GE (2005). Mitochondrial abnormalities in Alzheimer brain: mechanistic implications. Ann Neurol.

[52] Mastroeni D, Khdour OM, Delvaux E, Nolz J, Olsen G, Berchtold N, Cotman C, Hecht SM, Coleman PD (2017). Nuclear but not mitochondrial-encoded oxidative phosphorylation genes are altered in aging, mild cognitive impairment, and Alzheimer's disease. Alzheimer's Dementia.

[53] Zhang L, Guo XQ, Chu JF, Zhang X, Yan ZR, Li YZ (2015). Potential hippocampal genes and pathways involved in Alzheimer's disease: a bioinformatic analysis. Genet Mol Res (GMR).

[54] Beck JS, Mufson EJ, Counts SE (2016). Evidence for mitochondrial UPR gene activation in familial and sporadic Alzheimer's disease. Curr Alzheimer Res.

[55] Gibson GE, Park LC, Zhang H, Sorbi S, Calingasan NY (1999). Oxidative stress and a key metabolic enzyme in Alzheimer brains, cultured cells, and an animal model of chronic oxidative deficits. Ann NY Acad Sci.

[56] Arslan J, Jamshed H, Qureshi H (2020). Early detection and prevention of Alzheimer's Disease: role of oxidative markers and natural antioxidants. Front Aging Neurosci.

[57] Au HC, Scheffler IE (1998). Promoter analysis of the human succinate dehydrogenase iron-protein gene–both nuclear respiratory factors NRF-1 and NRF-2 are required. Eur J Biochem.

[58] Miranda S, Foncea R, Guerrero J, Leighton F (1999). Oxidative stress and upregulation of mitochondrial biogenesis genes in mitochondrial DNA-depleted HeLa cells. Biochem Biophys Res Commun.

[59] Shi Q, Gibson GE (2011). Up-regulation of the mitochondrial malate dehydrogenase by oxidative stress is mediated by miR-743a. J Neurochem.

[60] Varoqui H, Erickson JD (1996). Active transport of acetylcholine by the human vesicular acetylcholine transporter. J Biol Chem.

[61] Bierer LM, Haroutunian V, Gabriel S, Knott PJ, Carlin LS, Purohit DP, Perl DP, Schmeidler J, Kanof P, Davis KL (1995). Neurochemical correlates of dementia severity in Alzheimer's disease: relative importance of the cholinergic deficits. J Neurochem.

[62] Stanciu GD, Luca A, Rusu RN, Bild V, Beschea Chiriac SI, Solcan C, Bild W, Ababei DC (2019). Alzheimer's disease pharmacotherapy in relation to cholinergic system involvement. Biomolecules.

[63] Denton RM (2009). Regulation of mitochondrial dehydrogenases by calcium ions. Biochim Biophys Acta.

[64] Denton RM, Randle PJ, Martin BR (1972). Stimulation by calcium ions of pyruvate dehydrogenase phosphate phosphatase. Biochem J.

[65] Denton RM, Richards DA, Chin JG (1978). Calcium ions and the regulation of NAD+-linked isocitrate dehydrogenase from the mitochondria of rat heart and other tissues. Biochem J.

[66] Sabatini BL, Regehr WG (1996). Timing of neurotransmission at fast synapses in the mammalian brain. Nature.

[67] Sudhof TC (2012). Calcium control of neurotransmitter release. Cold Spring Harb Perspect Biol.

[68] Verstreken P, Ly CV, Venken KJ, Koh TW, Zhou Y, Bellen HJ (2005). Synaptic mitochondria are critical for mobilization of reserve pool vesicles at Drosophila neuromuscular junctions. Neuron.

[69] Berridge MJ (1998). Neuronal calcium signaling. Neuron.

[70] Genovese I, Giamogante F, Barazzuol L, Battista T, Fiorillo A, Vicario M, D'Alessandro G, Cipriani R, Limatola C, Rossi D (2020). Sorcin is an early marker of neurodegeneration, Ca(2+) dysregulation and endoplasmic reticulum stress associated to neurodegenerative diseases. Cell Death Dis.

[71] Baughman JM, Perocchi F, Girgis HS, Plovanich M, Belcher-Timme CA, Sancak Y, Bao XR, Strittmatter L, Goldberger O, Bogorad RL (2011). Integrative genomics identifies MCU as an essential component of the mitochondrial calcium uniporter. Nature.

[72] De Stefani D, Raffaello A, Teardo E, Szabo I, Rizzuto R (2011). A forty-kilodalton protein of the inner membrane is the mitochondrial calcium uniporter. Nature.

[73] Luongo TS, Lambert JP, Gross P, Nwokedi M, Lombardi AA, Shanmughapriya S, Carpenter AC, Kolmetzky D, Gao E, van Berlo JH (2017). The mitochondrial Na(+)/Ca(2+) exchanger is essential for Ca(2+) homeostasis and viability. Nature.

[74] Palty R, Silverman WF, Hershfinkel M, Caporale T, Sensi SL, Parnis J, Nolte C, Fishman D, Shoshan-Barmatz V, Herrmann S (2010). NCLX is an essential component of mitochondrial Na+/Ca2+ exchange. Proc Natl Acad Sci USA.

[75] Calvo-Rodriguez M, Hou SS, Snyder AC, Kharitonova EK, Russ AN, Das S, Fan Z, Muzikansky A, Garcia-Alloza M, Serrano-Pozo A (2020). Increased mitochondrial calcium levels associated with neuronal death in a mouse model of Alzheimer's disease. Nat Commun.

[76] Granatiero V, Pacifici M, Raffaello A, De Stefani D, Rizzuto R (2019). Overexpression of mitochondrial calcium uniporter causes neuronal death. Oxid Med Cell Longev.

[77] Jadiya P, Kolmetzky DW, Tomar D, Di Meco A, Lombardi AA, Lambert JP, Luongo TS, Ludtmann MH, Pratico D, Elrod JW (2019). Impaired mitochondrial calcium efflux contributes to disease progression in models of Alzheimer's disease. Nat Commun.

[78] Qiu J, Tan YW, Hagenston AM, Martel MA, Kneisel N, Skehel PA, Wyllie DJ, Bading H, Hardingham GE (2013). Mitochondrial calcium uniporter Mcu controls excitotoxicity and is transcriptionally repressed by neuroprotective nuclear calcium signals. Nat Commun.

[79] Chou JL, Shenoy DV, Thomas N, Choudhary PK, Laferla FM, Goodman SR, Breen GA (2011). Early dysregulation of the mitochondrial proteome in a mouse model of Alzheimer's disease. J Proteomics.

[80] Volgyi K, Badics K, Sialana FJ, Gulyassy P, Udvari EB, Kis V, Drahos L, Lubec G, Kekesi KA, Juhasz G (2018). Early presymptomatic changes in the proteome of mitochondria-associated membrane in the APP/PS1 mouse model of Alzheimer's disease. Mol Neurobiol.

[81] Evans AR, Gu L, Guerrero R, Robinson RA (2015). Global cPILOT analysis of the APP/PS-1 mouse liver proteome. Proteomics Clin Appl.

[82] Lewis TL, Kwon SK, Lee A, Shaw R, Polleux F (2018). MFF-dependent mitochondrial fission regulates presynaptic release and axon branching by limiting axonal mitochondria size. Nat Commun.

[83] Mandal A, Drerup CM (2019). Axonal transport and mitochondrial function in neurons. Front Cell Neurosci.

[84] Saxton WM, Hollenbeck PJ (2012). The axonal transport of mitochondria. J Cell Sci.

[85] Hirokawa N, Takemura R (2005). Molecular motors and mechanisms of directional transport in neurons. Nat Rev Neurosci.

[86] Sleigh JN, Rossor AM, Fellows AD, Tosolini AP, Schiavo G (2019). Axonal transport and neurological disease. Nat Rev Neurol.

[87] Stokin GB, Lillo C, Falzone TL, Brusch RG, Rockenstein E, Mount SL, Raman R, Davies P, Masliah E, Williams DS (2005). Axonopathy and transport deficits early in the pathogenesis of Alzheimer's disease. Science.

[88] Cai Q, Tammineni P (2016). Alterations in mitochondrial quality control in Alzheimer's disease. Front Cell Neurosci.

[89] Wang X, Schwarz TL (2009). The mechanism of Ca2+ -dependent regulation of kinesin-mediated mitochondrial motility. Cell.

[90] Chang KT, Niescier RF, Min KT (2011). Mitochondrial matrix Ca2+ as an intrinsic signal regulating mitochondrial motility in axons. Proc Natl Acad Sci USA.

[91] Niescier RF, Hong K, Park D, Min KT (2018). MCU interacts with Miro1 to modulate mitochondrial functions in neurons. J Neurosci Off J Soc Neurosci.

[92] Dorsey ER, Bloem BR (2018). The Parkinson Pandemic-a call to action. JAMA Neurol.

[93] Simon-Gozalbo A, Rodriguez-Blazquez C, Forjaz MJ, Martinez-Martin P (2020). Clinical characterization of Parkinson's disease patients with cognitive impairment. Front Neurol.

[94] Polymeropoulos MH, Lavedan C, Leroy E, Ide SE, Dehejia A, Dutra A, Pike B, Root H, Rubenstein J, Boyer R (1997). Mutation in the alpha-synuclein gene identified in families with Parkinson's disease. Science.

[95] Kitada T, Asakawa S, Hattori N, Matsumine H, Yamamura Y, Minoshima S, Yokochi M, Mizuno Y, Shimizu N (1998). Mutations in the parkin gene cause autosomal recessive juvenile parkinsonism. Nature.

[96] Bonifati V, Rizzu P, van Baren MJ, Schaap O, Breedveld GJ, Krieger E, Dekker MC, Squitieri F, Ibanez P, Joosse M (2003). Mutations in the DJ-1 gene associated with autosomal recessive early-onset parkinsonism. Science.

[97] Zimprich A, Biskup S, Leitner P, Lichtner P, Farrer M, Lincoln S, Kachergus J, Hulihan M, Uitti RJ, Calne DB (2004). Mutations in LRRK2 cause autosomal-dominant parkinsonism with pleomorphic pathology. Neuron.

[98] Valente EM, Abou-Sleiman PM, Caputo V, Muqit MM, Harvey K, Gispert S, Ali Z, Del Turco D, Bentivoglio AR, Healy DG (2004). Hereditary early-onset Parkinson's disease caused by mutations in PINK1. Science.

[99] Dawson TM, Dawson VL (2010). The role of parkin in familial and sporadic Parkinson's disease. Mov Disord.

[100] LeWitt PA, Giladi N, Navon N (2019). Pharmacokinetics and efficacy of a novel formulation of carbidopa-levodopa (Accordion Pill((R))) in Parkinson's disease. Parkinsonism Relat Disord.

[101] LeWitt PA, Hauser RA, Pahwa R, Isaacson SH, Fernandez HH, Lew M, Saint-Hilaire M, Pourcher E, Lopez-Manzanares L, Waters C (2019). Safety and efficacy of CVT-301 (levodopa inhalation powder) on motor function during off periods in patients with Parkinson's disease: a randomised, double-blind, placebo-controlled phase 3 trial. Lancet Neurol.

[102] Muller T, Mohr JD (2019). Recent clinical advances in pharmacotherapy for levodopa-induced dyskinesia. Drugs.

[103] Espay AJ, Kalia LV, Gan-Or Z, Williams-Gray CH, Bedard PL, Rowe SM, Morgante F, Fasano A, Stecher B, Kauffman MA (2020). Disease modification and biomarker development in Parkinson disease: revision or reconstruction?. Neurology.

[104] Simuni T, Siderowf A, Lasch S, Coffey CS, Caspell-Garcia C, Jennings D, Tanner CM, Trojanowski JQ, Shaw LM, Seibyl J (2018). Longitudinal change of clinical and biological measures in early Parkinson's disease: Parkinson's progression markers initiative cohort. Mov Disord.

[105] Jansen IE, Ye H, Heetveld S, Lechler MC, Michels H, Seinstra RI, Lubbe SJ, Drouet V, Lesage S, Majounie E (2017). Discovery and functional prioritization of Parkinson's disease candidate genes from large-scale whole exome sequencing. Genome Biol.

[106] Milanese C, Payan-Gomez C, Galvani M, Molano Gonzalez N, Tresini M, Nait Abdellah S, van Roon-Mom WMC, Figini S, Marinus J, van Hilten JJ (2019). Peripheral mitochondrial function correlates with clinical severity in idiopathic Parkinson's disease. Mov Disord.

[107] Mamelak M (2018). Parkinson's disease, the dopaminergic neuron and gammahydroxybutyrate. Neurol Ther.

[108] Juarez Olguin H, Calderon Guzman D, Hernandez Garcia E, Barragan Mejia G (2016). The role of dopamine and its dysfunction as a consequence of oxidative stress. Oxid Med Cell Longev.

[109] Kim TY, Leem E, Lee JM, Kim SR (2020). Control of reactive oxygen species for the prevention of Parkinson's disease: the possible application of flavonoids. Antioxidants (Basel).

[110] Martin WR, Hayden MR (1987). Cerebral glucose and dopa metabolism in movement disorders. Can J Neurol Sci.

[111] Mytilineou C, Werner P, Molinari S, Di Rocco A, Cohen G, Yahr MD (1994). Impaired oxidative decarboxylation of pyruvate in fibroblasts from patients with Parkinson's disease. J Neural Transm Park Dis Dement Sect.

[112] Langston JW, Ballard P, Tetrud JW, Irwin I (1983). Chronic Parkinsonism in humans due to a product of meperidine-analog synthesis. Science.

[113] Schapira AH, Cooper JM, Dexter D, Jenner P, Clark JB, Marsden CD (1989). Mitochondrial complex I deficiency in Parkinson's disease. Lancet.

[114] Perier C, Vila M (2012). Mitochondrial biology and Parkinson's disease. Cold Spring Harb Perspect Med.

[115] Cannon JR, Greenamyre JT (2010). Neurotoxic in vivo models of Parkinson's disease recent advances. Prog Brain Res.

[116] Jackson-Lewis V, Przedborski S (2007). Protocol for the MPTP mouse model of Parkinson's disease. Nat Protoc.

[117] Valdez LB, Zaobornyj T, Bandez MJ, Lopez-Cepero JM, Boveris A, Navarro A (2019). Complex I syndrome in striatum and frontal cortex in a rat model of Parkinson disease. Free Radic Biol Med.

[118] Galkin A, Drose S, Brandt U (2006). The proton pumping stoichiometry of purified mitochondrial complex I reconstituted into proteoliposomes. Biochim Biophys Acta.

[119] Wikstrom M (1984). Two protons are pumped from the mitochondrial matrix per electron transferred between NADH and ubiquinone. FEBS Lett.

[120] Grunewald A, Rygiel KA, Hepplewhite PD, Morris CM, Picard M, Turnbull DM (2016). Mitochondrial DNA depletion in respiratory chain-deficient Parkinson disease neurons. Ann Neurol.

[121] Haas RH, Nasirian F, Nakano K, Ward D, Pay M, Hill R, Shults CW (1995). Low platelet mitochondrial complex I and complex II/III activity in early untreated Parkinson's disease. Ann Neurol.

[122] Shinde S, Pasupathy K (2006). Respiratory-chain enzyme activities in isolated mitochondria of lymphocytes from patients with Parkinson's disease: preliminary study. Neurol India.

[123] Chan P, DeLanney LE, Irwin I, Langston JW, Di Monte D (1991). Rapid ATP loss caused by 1-methyl-4-phenyl-1,2,3,6-tetrahydropyridine in mouse brain. J Neurochem.

[124] Anne C, Gasnier B (2014). Vesicular neurotransmitter transporters: mechanistic aspects. Curr Top Membr.

[125] Lotharius J, Brundin P (2002). Impaired dopamine storage resulting from alpha-synuclein mutations may contribute to the pathogenesis of Parkinson's disease. Hum Mol Genet.

[126] Chu Y, Morfini GA, Langhamer LB, He Y, Brady ST, Kordower JH (2012). Alterations in axonal transport motor proteins in sporadic and experimental Parkinson's disease. Brain.

[127] Han H, Tan J, Wang R, Wan H, He Y, Yan X, Guo J, Gao Q, Li J, Shang S (2020). PINK1 phosphorylates Drp 1(S616) to regulate mitophagy-independent mitochondrial dynamics. EMBO Rep.

[128] Van Laar VS, Berman SB (2013). The interplay of neuronal mitochondrial dynamics and bioenergetics: implications for Parkinson's disease. Neurobiol Dis.

[129] De Mattos EP, Wentink A, Nussbaum-Krammer C, Hansen C, Bergink S, Melki R, Kampinga HH (2020). Protein quality control pathways at the crossroad of synucleinopathies. J Parkinsons Dis.

[130] Pantazopoulou M, Brembati V, Kanellidi A, Bousset L, Melki R, Stefanis L (2020). Distinct alpha-Synuclein species induced by seeding are selectively cleared by the Lysosome or the Proteasome in neuronally differentiated SH-SY5Y cells. J Neurochem.

[131] Musgrove RE, Helwig M, Bae EJ, Aboutalebi H, Lee SJ, Ulusoy A, Di Monte DA (2019). Oxidative stress in vagal neurons promotes parkinsonian pathology and intercellular alpha-synuclein transfer. J Clin Investig.

[132] Perier C, Tieu K, Guegan C, Caspersen C, Jackson-Lewis V, Carelli V, Martinuzzi A, Hirano M, Przedborski S, Vila M (2005). Complex I deficiency primes Bax-dependent neuronal apoptosis through mitochondrial oxidative damage. Proc Natl Acad Sci USA.

[133] Tapias V, McCoy JL, Greenamyre JT (2019). Phenothiazine normalizes the NADH/NAD(+) ratio, maintains mitochondrial integrity and protects the nigrostriatal dopamine system in a chronic rotenone model of Parkinson's disease. Redox Biol.

[134] Kristal BS, Conway AD, Brown AM, Jain JC, Ulluci PA, Li SW, Burke WJ (2001). Selective dopaminergic vulnerability: 3,4-dihydroxyphenylacetaldehyde targets mitochondria. Free Radic Biol Med.

[135] Parihar MS, Parihar A, Fujita M, Hashimoto M, Ghafourifar P (2008). Mitochondrial association of alpha-synuclein causes oxidative stress. Cell Mol Life Sci (CMLS).

[136] Hu D, Sun X, Liao X, Zhang X, Zarabi S, Schimmer A, Hong Y, Ford C, Luo Y, Qi X (2019). Alpha-synuclein suppresses mitochondrial protease ClpP to trigger mitochondrial oxidative damage and neurotoxicity. Acta Neuropathol.

[137] Faustini G, Bono F, Valerio A, Pizzi M, Spano P, Bellucci A (2017). Mitochondria and alpha-Synuclein: friends or foes in the pathogenesis of Parkinson's disease?. Genes.

[138] Mahul-Mellier AL, Burtscher J, Maharjan N, Weerens L, Croisier M, Kuttler F, Leleu M, Knott GW, Lashuel HA (2020). The process of Lewy body formation, rather than simply alpha-synuclein fibrillization, is one of the major drivers of neurodegeneration. Proc Natl Acad Sci USA.

[139] Chen R, Park HA, Mnatsakanyan N, Niu Y, Licznerski P, Wu J, Miranda P, Graham M, Tang J, Boon AJW (2019). Parkinson's disease protein DJ-1 regulates ATP synthase protein components to increase neuronal process outgrowth. Cell Death Dis.

[140] Heo JY, Park JH, Kim SJ, Seo KS, Han JS, Lee SH, Kim JM, Park JI, Park SK, Lim K (2012). DJ-1 null dopaminergic neuronal cells exhibit defects in mitochondrial function and structure: involvement of mitochondrial complex I assembly. PLoS ONE.

[141] Bian M, Liu J, Hong X, Yu M, Huang Y, Sheng Z, Fei J, Huang F (2012). Overexpression of parkin ameliorates dopaminergic neurodegeneration induced by 1- methyl-4-phenyl-1,2,3,6-tetrahydropyridine in mice. PLoS ONE.

[142] Chung E, Choi Y, Park J, Nah W, Park J, Jung Y, Lee J, Lee H, Park S, Hwang S (2020). Intracellular delivery of Parkin rescues neurons from accumulation of damaged mitochondria and pathological alpha-synuclein. Sci Adv.

[143] Darios F, Corti O, Lucking CB, Hampe C, Muriel MP, Abbas N, Gu WJ, Hirsch EC, Rooney T, Ruberg M (2003). Parkin prevents mitochondrial swelling and cytochrome c release in mitochondria-dependent cell death. Hum Mol Genet.

[144] Ge P, Dawson VL, Dawson TM (2020). PINK1 and Parkin mitochondrial quality control: a source of regional vulnerability in Parkinson's disease. Mol Neurodegener.

[145] Narendra D, Tanaka A, Suen DF, Youle RJ (2008). Parkin is recruited selectively to impaired mitochondria and promotes their autophagy. J Cell Biol.

[146] Gandhi S, Wood-Kaczmar A, Yao Z, Plun-Favreau H, Deas E, Klupsch K, Downward J, Latchman DS, Tabrizi SJ, Wood NW (2009). PINK1-associated Parkinson's disease is caused by neuronal vulnerability to calcium-induced cell death. Mol Cell.

[147] Abramov AY, Gegg M, Grunewald A, Wood NW, Klein C, Schapira AH (2011). Bioenergetic consequences of PINK1 mutations in Parkinson disease. PLoS ONE.

[148] Kostic M, Ludtmann MH, Bading H, Hershfinkel M, Steer E, Chu CT, Abramov AY, Sekler I (2015). PKA phosphorylation of NCLX reverses mitochondrial calcium overload and depolarization, promoting survival of PINK1-deficient dopaminergic neurons. Cell Rep.

[149] Verma M, Callio J, Otero PA, Sekler I, Wills ZP, Chu CT (2017). Mitochondrial calcium dysregulation contributes to dendrite degeneration mediated by PD/LBD-associated LRRK2 mutants. J Neurosci Off J Soc Neurosci.

[150] Soman S, Keatinge M, Moein M, Da Costa M, Mortiboys H, Skupin A, Sugunan S, Bazala M, Kuznicki J, Bandmann O (2017). Inhibition of the mitochondrial calcium uniporter rescues dopaminergic neurons in pink1(-/-) zebrafish. Eur J Neurosci.

[151] Soman SK, Bazala M, Keatinge M, Bandmann O, Kuznicki J (2019). Restriction of mitochondrial calcium overload by mcu inactivation renders a neuroprotective effect in zebrafish models of Parkinson's disease. Biol Open.

[152] Anil M, Mason SL, Barker RA (2020). The clinical features and progression of late-onset versus younger-onset in an adult cohort of huntington's disease patients. J Huntington's Dis.

[153] Kuhl DE, Phelps ME, Markham CH, Metter EJ, Riege WH, Winter J (1982). Cerebral metabolism and atrophy in Huntington's disease determined by 18FDG and computed tomographic scan. Ann Neurol.

[154] Sanberg PR, Fibiger HC, Mark RF (1981). Body weight and dietary factors in Huntington's disease patients compared with matched controls. Med J Aust.

[155] Berent S, Giordani B, Lehtinen S, Markel D, Penney JB, Buchtel HA, Starosta-Rubinstein S, Hichwa R, Young AB (1988). Positron emission tomographic scan investigations of Huntington's disease: cerebral metabolic correlates of cognitive function. Ann Neurol.

[156] Morea V, Bidollari E, Colotti G, Fiorillo A, Rosati J, De Filippis L, Squitieri F, Ilari A (2017). Glucose transportation in the brain and its impairment in Huntington disease: one more shade of the energetic metabolism failure?. Amino Acids.

[157] Lee JK, Mathews K, Schlaggar B, Perlmutter J, Paulsen JS, Epping E, Burmeister L, Nopoulos P (2012). Measures of growth in children at risk for Huntington disease. Neurology.

[158] Naseri NN, Bonica J, Xu H, Park LC, Arjomand J, Chen Z, Gibson GE (2016). Novel metabolic abnormalities in the tricarboxylic acid cycle in peripheral cells from Huntington's disease patients. PLoS ONE.

[159] Ciammola A, Sassone J, Sciacco M, Mencacci NE, Ripolone M, Bizzi C, Colciago C, Moggio M, Parati G, Silani V (2011). Low anaerobic threshold and increased skeletal muscle lactate production in subjects with Huntington's disease. Mov Disord.

[160] Jenkins BG, Koroshetz WJ, Beal MF, Rosen BR (1993). Evidence for impairment of energy metabolism in vivo in Huntington's disease using localized 1H NMR spectroscopy. Neurology.

[161] Jenkins BG, Rosas HD, Chen YC, Makabe T, Myers R, MacDonald M, Rosen BR, Beal MF, Koroshetz WJ (1998). 1H NMR spectroscopy studies of Huntington's disease: correlations with CAG repeat numbers. Neurology.

[162] Martin WR, Wieler M, Hanstock CC (2007). Is brain lactate increased in Huntington's disease?. J Neurol Sci.

[163] Beal MF, Brouillet E, Jenkins BG, Ferrante RJ, Kowall NW, Miller JM, Storey E, Srivastava R, Rosen BR, Hyman BT (1993). Neurochemical and histologic characterization of striatal excitotoxic lesions produced by the mitochondrial toxin 3-nitropropionic acid. J Neurosci Off J Soc Neurosci.

[164] Brouillet E, Hantraye P, Ferrante RJ, Dolan R, Leroy-Willig A, Kowall NW, Beal MF (1995). Chronic mitochondrial energy impairment produces selective striatal degeneration and abnormal choreiform movements in primates. Proc Natl Acad Sci USA.

[165] Wiprich MT, Zanandrea R, Altenhofen S, Bonan CD (2020). Influence of 3-nitropropionic acid on physiological and behavioral responses in zebrafish larvae and adults. Comp Biochem Physiol C Toxicol Pharmacol.

[166] Andreassen OA, Dedeoglu A, Ferrante RJ, Jenkins BG, Ferrante KL, Thomas M, Friedlich A, Browne SE, Schilling G, Borchelt DR (2001). Creatine increase survival and delays motor symptoms in a transgenic animal model of Huntington's disease. Neurobiol Dis.

[167] Yang L, Calingasan NY, Wille EJ, Cormier K, Smith K, Ferrante RJ, Beal MF (2009). Combination therapy with coenzyme Q10 and creatine produces additive neuroprotective effects in models of Parkinson's and Huntington's diseases. J Neurochem.

[168] Lopes C, Tang Y, Anjo SI, Manadas B, Onofre I, de Almeida LP, Daley GQ, Schlaeger TM, Rego ACC (2020). Mitochondrial and redox modifications in huntington disease induced pluripotent stem cells rescued by CRISPR/Cas9 CAGs targeting. Front Cell Dev Biol.

[169] McQuade LR, Balachandran A, Scott HA, Khaira S, Baker MS, Schmidt U (2014). Proteomics of Huntington's disease-affected human embryonic stem cells reveals an evolving pathology involving mitochondrial dysfunction and metabolic disturbances. J Proteome Res.

[170] Panov AV, Gutekunst CA, Leavitt BR, Hayden MR, Burke JR, Strittmatter WJ, Greenamyre JT (2002). Early mitochondrial calcium defects in Huntington's disease are a direct effect of polyglutamines. Nat Neurosci.

[171] Cherubini M, Lopez-Molina L, Gines S (2020). Mitochondrial fission in Huntington's disease mouse striatum disrupts ER-mitochondria contacts leading to disturbances in Ca(2+) efflux and Reactive Oxygen Species (ROS) homeostasis. Neurobiol Dis.

[172] Gunawardena S, Her LS, Brusch RG, Laymon RA, Niesman IR, Gordesky-Gold B, Sintasath L, Bonini NM, Goldstein LS (2003). Disruption of axonal transport by loss of huntingtin or expression of pathogenic polyQ proteins in Drosophila. Neuron.

[173] Area-Gomez E, Guardia-Laguarta C, Schon EA, Przedborski S (2019). Mitochondria, OxPhos, and neurodegeneration: cells are not just running out of gas. J Clin Investig.

[174] Camandola S, Mattson MP (2017). Brain metabolism in health, aging, and neurodegeneration. EMBO J.

[175] Pchitskaya E, Popugaeva E, Bezprozvanny I (2018). Calcium signaling and molecular mechanisms underlying neurodegenerative diseases. Cell Calcium.

[176] Mattson MP (2007). Calcium and neurodegeneration. Aging Cell.

[177] Popugaeva E, Pchitskaya E, Bezprozvanny I (2018). Dysregulation of intracellular calcium signaling in Alzheimer's disease. Antioxid Redox Signal.

[178] Popugaeva E, Pchitskaya E, Bezprozvanny I (2017). Dysregulation of neuronal calcium homeostasis in Alzheimer's disease—a therapeutic opportunity?. Biochem Biophys Res Commun.

[179] Hypothesis AAC, W, (2017). Calcium Hypothesis of Alzheimer's disease and brain aging: a framework for integrating new evidence into a comprehensive theory of pathogenesis. Alzheimer's Dementia.

[180] Bezprozvanny I, Mattson MP (2008). Neuronal calcium mishandling and the pathogenesis of Alzheimer's disease. Trends Neurosci.

[181] Mattson MP, Cheng B, Davis D, Bryant K, Lieberburg I, Rydel RE (1992). beta-Amyloid peptides destabilize calcium homeostasis and render human cortical neurons vulnerable to excitotoxicity. J Neurosci Off J Soc Neurosci.

[182] Arispe N, Diaz JC, Simakova O (2007). Abeta ion channels. prospects for treating Alzheimer's disease with Abeta channel blockers. Biochim Biophys Acta.

[183] Kuchibhotla KV, Goldman ST, Lattarulo CR, Wu HY, Hyman BT, Bacskai BJ (2008). Abeta plaques lead to aberrant regulation of calcium homeostasis in vivo resulting in structural and functional disruption of neuronal networks. Neuron.

[184] Ueda K, Shinohara S, Yagami T, Asakura K, Kawasaki K (1997). Amyloid beta protein potentiates Ca2+ influx through L-type voltage-sensitive Ca2+ channels: a possible involvement of free radicals. J Neurochem.

[185] Nimmrich V, Grimm C, Draguhn A, Barghorn S, Lehmann A, Schoemaker H, Hillen H, Gross G, Ebert U, Bruehl C (2008). Amyloid beta oligomers (A beta(1–42) globulomer) suppress spontaneous synaptic activity by inhibition of P/Q-type calcium currents. J Neurosci Off J Soc Neurosci.

[186] Zhang Y, Li P, Feng J, Wu M (2016). Dysfunction of NMDA receptors in Alzheimer's disease. Neurol Sci Off J Ital Neurol Soc Ital Soc Clin Neurophysiol.

[187] Zaichick SV, McGrath KM, Caraveo G (2017). The role of Ca(2+) signaling in Parkinson's disease. Dis Model Mech.

[188] Furukawa K, Matsuzaki-Kobayashi M, Hasegawa T, Kikuchi A, Sugeno N, Itoyama Y, Wang Y, Yao PJ, Bushlin I, Takeda A (2006). Plasma membrane ion permeability induced by mutant alpha-synuclein contributes to the degeneration of neural cells. J Neurochem.

[189] Ilijic E, Guzman JN, Surmeier DJ (2011). The L-type channel antagonist isradipine is neuroprotective in a mouse model of Parkinson's disease. Neurobiol Dis.

[190] Raymond LA (2017). Striatal synaptic dysfunction and altered calcium regulation in Huntington disease. Biochem Biophys Res Commun.

[191] Tang TS, Tu H, Chan EY, Maximov A, Wang Z, Wellington CL, Hayden MR, Bezprozvanny I (2003). Huntingtin and huntingtin-associated protein 1 influence neuronal calcium signaling mediated by inositol-(1,4,5) triphosphate receptor type 1. Neuron.

[192] Wu J, Ryskamp DA, Liang X, Egorova P, Zakharova O, Hung G, Bezprozvanny I (2016). Enhanced store-operated calcium entry leads to striatal synaptic loss in a Huntington's disease mouse model. J Neurosci Off J Soc Neurosci.

[193] Csordas G, Thomas AP, Hajnoczky G (1999). Quasi-synaptic calcium signal transmission between endoplasmic reticulum and mitochondria. EMBO J.

[194] Rizzuto R, Pinton P, Carrington W, Fay FS, Fogarty KE, Lifshitz LM, Tuft RA, Pozzan T (1998). Close contacts with the endoplasmic reticulum as determinants of mitochondrial Ca2+ responses. Science.

[195] Szabadkai G, Simoni AM, Rizzuto R (2003). Mitochondrial Ca2+ uptake requires sustained Ca2+ release from the endoplasmic reticulum. J Biol Chem.

[196] Dolphin AC, Lee A (2020). Presynaptic calcium channels: specialized control of synaptic neurotransmitter release. Nat Rev Neurosci.

[197] Pinton P, Giorgi C, Siviero R, Zecchini E, Rizzuto R (2008). Calcium and apoptosis: ER-mitochondria Ca2+ transfer in the control of apoptosis. Oncogene.

[198] Cali T, Ottolini D, Brini M (2012). Mitochondrial Ca(2+) and neurodegeneration. Cell Calcium.

[199] Ryan KC, Ashkavand Z, Norman KR (2020). The role of mitochondrial calcium homeostasis in Alzheimer's and related diseases. Int J Mol Sci.

[200] Calvo-Rodriguez M, Hernando-Perez E, Nunez L, Villalobos C (2019). Amyloid beta oligomers increase ER-mitochondria Ca(2+) cross talk in young hippocampal neurons and exacerbate aging-induced intracellular Ca(2+) remodeling. Front Cell Neurosci.

[201] Caspersen C, Wang N, Yao J, Sosunov A, Chen X, Lustbader JW, Xu HW, Stern D, McKhann G, Yan SD (2005). Mitochondrial Abeta: a potential focal point for neuronal metabolic dysfunction in Alzheimer's disease. FASEB J.

[202] Du H, Guo L, Fang F, Chen D, Sosunov AA, McKhann GM, Yan Y, Wang C, Zhang H, Molkentin JD (2008). Cyclophilin D deficiency attenuates mitochondrial and neuronal perturbation and ameliorates learning and memory in Alzheimer's disease. Nat Med.

[203] Marongiu R, Spencer B, Crews L, Adame A, Patrick C, Trejo M, Dallapiccola B, Valente EM, Masliah E (2009). Mutant Pink1 induces mitochondrial dysfunction in a neuronal cell model of Parkinson's disease by disturbing calcium flux. J Neurochem.

[204] Parihar MS, Parihar A, Fujita M, Hashimoto M, Ghafourifar P (2009). Alpha-synuclein overexpression and aggregation exacerbates impairment of mitochondrial functions by augmenting oxidative stress in human neuroblastoma cells. Int J Biochem Cell Biol.

[205] Hettiarachchi NT, Parker A, Dallas ML, Pennington K, Hung CC, Pearson HA, Boyle JP, Robinson P, Peers C (2009). alpha-Synuclein modulation of Ca2+ signaling in human neuroblastoma (SH-SY5Y) cells. J Neurochem.

[206] Cali T, Ottolini D, Negro A, Brini M (2012). alpha-Synuclein controls mitochondrial calcium homeostasis by enhancing endoplasmic reticulum-mitochondria interactions. J Biol Chem.

[207] Gomez-Suaga P, Bravo-San Pedro JM, Gonzalez-Polo RA, Fuentes JM, Niso-Santano M (2018). ER-mitochondria signaling in Parkinson's disease. Cell Death Dis.

[208] Flis VV, Daum G (2013). Lipid transport between the endoplasmic reticulum and mitochondria. Cold Spring Harb Perspect Biol.

[209] Joshi AS, Thompson MN, Fei N, Huttemann M, Greenberg ML (2012). Cardiolipin and mitochondrial phosphatidylethanolamine have overlapping functions in mitochondrial fusion in Saccharomyces cerevisiae. J Biol Chem.

[210] Friedman JR, Lackner LL, West M, DiBenedetto JR, Nunnari J, Voeltz GK (2011). ER tubules mark sites of mitochondrial division. Science.

[211] Schon EA, Area-Gomez E (2013). Mitochondria-associated ER membranes in Alzheimer disease. Mol Cell Neurosci.

[212] Bockler S, Westermann B (2014). ER-mitochondria contacts as sites of mitophagosome formation. Autophagy.

[213] Mironov SL, Symonchuk N (2006). ER vesicles and mitochondria move and communicate at synapses. J Cell Sci.

[214] Rangaraju V, Calloway N, Ryan TA (2014). Activity-driven local ATP synthesis is required for synaptic function. Cell.

[215] Achleitner G, Gaigg B, Krasser A, Kainersdorfer E, Kohlwein SD, Perktold A, Zellnig G, Daum G (1999). Association between the endoplasmic reticulum and mitochondria of yeast facilitates interorganelle transport of phospholipids through membrane contact. Eur J Biochem.

[216] Csordas G, Renken C, Varnai P, Walter L, Weaver D, Buttle KF, Balla T, Mannella CA, Hajnoczky G (2006). Structural and functional features and significance of the physical linkage between ER and mitochondria. J Cell Biol.

[217] Kornmann B, Currie E, Collins SR, Schuldiner M, Nunnari J, Weissman JS, Walter P (2009). An ER-mitochondria tethering complex revealed by a synthetic biology screen. Science.

[218] Herrera-Cruz MS, Simmen T (2017). Over six decades of discovery and characterization of the architecture at mitochondria-associated membranes (MAMs). Adv Exp Med Biol.

[219] Hirabayashi Y, Kwon SK, Paek H, Pernice WM, Paul MA, Lee J, Erfani P, Raczkowski A, Petrey DS, Pon LA (2017). ER-mitochondria tethering by PDZD8 regulates Ca(2+) dynamics in mammalian neurons. Science.

[220] de Brito OM, Scorrano L (2008). Mitofusin 2 tethers endoplasmic reticulum to mitochondria. Nature.

[221] Cosson P, Marchetti A, Ravazzola M, Orci L (2012). Mitofusin-2 independent juxtaposition of endoplasmic reticulum and mitochondria: an ultrastructural study. PLoS ONE.

[222] Leal NS, Schreiner B, Pinho CM, Filadi R, Wiehager B, Karlstrom H, Pizzo P, Ankarcrona M (2016). Mitofusin-2 knockdown increases ER-mitochondria contact and decreases amyloid beta-peptide production. J Cell Mol Med.

[223] Galmes R, Houcine A, van Vliet AR, Agostinis P, Jackson CL, Giordano F (2016). ORP5/ORP8 localize to endoplasmic reticulum-mitochondria contacts and are involved in mitochondrial function. EMBO Rep.

[224] Hung V, Lam SS, Udeshi ND, Svinkina T, Guzman G, Mootha VK, Carr SA, Ting AY (2017). Proteomic mapping of cytosol-facing outer mitochondrial and ER membranes in living human cells by proximity biotinylation. Elife.

[225] De Vos KJ, Morotz GM, Stoica R, Tudor EL, Lau KF, Ackerley S, Warley A, Shaw CE, Miller CC (2012). VAPB interacts with the mitochondrial protein PTPIP51 to regulate calcium homeostasis. Hum Mol Genet.

[226] Stoica R, De Vos KJ, Paillusson S, Mueller S, Sancho RM, Lau KF, Vizcay-Barrena G, Lin WL, Xu YF, Lewis J (2014). ER-mitochondria associations are regulated by the VAPB-PTPIP51 interaction and are disrupted by ALS/FTD-associated TDP-43. Nat Commun.

[227] Giorgi C, Missiroli S, Patergnani S, Duszynski J, Wieckowski MR, Pinton P (2015). Mitochondria-associated membranes: composition, molecular mechanisms, and physiopathological implications. Antioxid Redox Signal.

[228] Rodriguez-Arribas M, Yakhine-Diop SMS, Pedro JMB, Gomez-Suaga P, Gomez-Sanchez R, Martinez-Chacon G, Fuentes JM, Gonzalez-Polo RA, Niso-Santano M (2017). Mitochondria-associated membranes (MAMs): overview and its role in Parkinson's disease. Mol Neurobiol.

[229] Cardenas C, Miller RA, Smith I, Bui T, Molgo J, Muller M, Vais H, Cheung KH, Yang J, Parker I (2010). Essential regulation of cell bioenergetics by constitutive InsP3 receptor Ca2+ transfer to mitochondria. Cell.

[230] Gomez-Suaga P, Paillusson S, Stoica R, Noble W, Hanger DP, Miller CCJ (2017). The ER-mitochondria tethering complex VAPB-PTPIP51 regulates autophagy. Curr Biol.

[231] van Vliet AR, Verfaillie T, Agostinis P (2014). New functions of mitochondria associated membranes in cellular signaling. Biochim Biophys Acta.

[232] Vance JE (2014). MAM (mitochondria-associated membranes) in mammalian cells: lipids and beyond. Biochim Biophys Acta.

[233] Acehan D, Malhotra A, Xu Y, Ren M, Stokes DL, Schlame M (2011). Cardiolipin affects the supramolecular organization of ATP synthase in mitochondria. Biophys J.

[234] Gohil VM, Hayes P, Matsuyama S, Schagger H, Schlame M, Greenberg ML (2004). Cardiolipin biosynthesis and mitochondrial respiratory chain function are interdependent. J Biol Chem.

[235] Pfeiffer K, Gohil V, Stuart RA, Hunte C, Brandt U, Greenberg ML, Schagger H (2003). Cardiolipin stabilizes respiratory chain supercomplexes. J Biol Chem.

[236] Schlame M, Rua D, Greenberg ML (2000). The biosynthesis and functional role of cardiolipin. Prog Lipid Res.

[237] Paillusson S, Stoica R, Gomez-Suaga P, Lau DHW, Mueller S, Miller T, Miller CCJ (2016). There's something wrong with my MAM; the ER-mitochondria Axis and neurodegenerative diseases. Trends Neurosci.

[238] Area-Gomez E, de Groof AJ, Boldogh I, Bird TD, Gibson GE, Koehler CM, Yu WH, Duff KE, Yaffe MP, Pon LA (2009). Presenilins are enriched in endoplasmic reticulum membranes associated with mitochondria. Am J Pathol.

[239] Marchi S, Patergnani S, Missiroli S, Morciano G, Rimessi A, Wieckowski MR, Giorgi C, Pinton P (2018). Mitochondrial and endoplasmic reticulum calcium homeostasis and cell death. Cell Calcium.

[240] Pera M, Larrea D, Guardia-Laguarta C, Montesinos J, Velasco KR, Agrawal RR, Xu Y, Chan RB, Di Paolo G, Mehler MF (2017). Increased localization of APP-C99 in mitochondria-associated ER membranes causes mitochondrial dysfunction in Alzheimer disease. EMBO J.

[241] Kogot-Levin A, Saada A (2014). Ceramide and the mitochondrial respiratory chain. Biochimie.

[242] Yu J, Novgorodov SA, Chudakova D, Zhu H, Bielawska A, Bielawski J, Obeid LM, Kindy MS, Gudz TI (2007). JNK3 signaling pathway activates ceramide synthase leading to mitochondrial dysfunction. J Biol Chem.

[243] Gautier CA, Erpapazoglou Z, Mouton-Liger F, Muriel MP, Cormier F, Bigou S, Duffaure S, Girard M, Foret B, Iannielli A (2016). The endoplasmic reticulum-mitochondria interface is perturbed in PARK2 knockout mice and patients with PARK2 mutations. Hum Mol Genet.

[244] Gelmetti V, De Rosa P, Torosantucci L, Marini ES, Romagnoli A, Di Rienzo M, Arena G, Vignone D, Fimia GM, Valente EM (2017). PINK1 and BECN1 relocalize at mitochondria-associated membranes during mitophagy and promote ER-mitochondria tethering and autophagosome formation. Autophagy.

[245] Paillusson S, Gomez-Suaga P, Stoica R, Little D, Gissen P, Devine MJ, Noble W, Hanger DP, Miller CCJ (2017). alpha-Synuclein binds to the ER-mitochondria tethering protein VAPB to disrupt Ca(2+) homeostasis and mitochondrial ATP production. Acta Neuropathol.

[246] Guardia-Laguarta C, Area-Gomez E, Rub C, Liu Y, Magrane J, Becker D, Voos W, Schon EA, Przedborski S (2014). alpha-Synuclein is localized to mitochondria-associated ER membranes. J Neurosci Off J Soc Neurosci.

[247] Bonifati V (2012). Autosomal recessive parkinsonism. Parkinsonism Relat Disord.

[248] Ottolini D, Cali T, Negro A, Brini M (2013). The Parkinson disease-related protein DJ-1 counteracts mitochondrial impairment induced by the tumour suppressor protein p53 by enhancing endoplasmic reticulum-mitochondria tethering. Hum Mol Genet.

[249] Liu Y, Ma X, Fujioka H, Liu J, Chen S, Zhu X (2019). DJ-1 regulates the integrity and function of ER-mitochondria association through interaction with IP3R3-Grp75-VDAC1. Proc Natl Acad Sci USA.

[250] Yang JY, Yang WY (2013). Bit-by-bit autophagic removal of parkin-labelled mitochondria. Nat Commun.

[251] Khacho M, Slack RS (2018). Mitochondrial dynamics in the regulation of neurogenesis: from development to the adult brain. Dev Dyn.

[252] Gomes LC, Di Benedetto G, Scorrano L (2011). During autophagy mitochondria elongate, are spared from degradation and sustain cell viability. Nat Cell Biol.

[253] Gao AW, Canto C, Houtkooper RH (2014). Mitochondrial response to nutrient availability and its role in metabolic disease. EMBO Mol Med.

[254] Li J, Wang Y, Wang Y, Wen X, Ma XN, Chen W, Huang F, Kou J, Qi LW, Liu B (2015). Pharmacological activation of AMPK prevents Drp1-mediated mitochondrial fission and alleviates endoplasmic reticulum stress-associated endothelial dysfunction. J Mol Cell Cardiol.

[255] Gao J, Wang L, Liu J, Xie F, Su B, Wang X (2017). Abnormalities of mitochondrial dynamics in neurodegenerative diseases. Antioxidants (Basel).

[256] Frank M, Duvezin-Caubet S, Koob S, Occhipinti A, Jagasia R, Petcherski A, Ruonala MO, Priault M, Salin B, Reichert AS (2012). Mitophagy is triggered by mild oxidative stress in a mitochondrial fission dependent manner. Biochim Biophys Acta.

[257] Hirai K, Aliev G, Nunomura A, Fujioka H, Russell RL, Atwood CS, Johnson AB, Kress Y, Vinters HV, Tabaton M (2001). Mitochondrial abnormalities in Alzheimer's disease. J Neurosci Off J Soc Neurosci.

[258] Reddy PH, McWeeney S, Park BS, Manczak M, Gutala RV, Partovi D, Jung Y, Yau V, Searles R, Mori M (2004). Gene expression profiles of transcripts in amyloid precursor protein transgenic mice: up-regulation of mitochondrial metabolism and apoptotic genes is an early cellular change in Alzheimer's disease. Hum Mol Genet.

[259] Diana A, Simic G, Sinforiani E, Orru N, Pichiri G, Bono G (2008). Mitochondria morphology and DNA content upon sublethal exposure to beta-amyloid(1–42) peptide. Coll Antropol.

[260] Chandrasekaran K, Giordano T, Brady DR, Stoll J, Martin LJ, Rapoport SI (1994). Impairment in mitochondrial cytochrome oxidase gene expression in Alzheimer disease. Brain Res Mol Brain Res.

[261] de la Monte SM, Luong T, Neely TR, Robinson D, Wands JR (2000). Mitochondrial DNA damage as a mechanism of cell loss in Alzheimer's disease. Lab Invest.

[262] Liang WS, Reiman EM, Valla J, Dunckley T, Beach TG, Grover A, Niedzielko TL, Schneider LE, Mastroeni D, Caselli R (2008). Alzheimer's disease is associated with reduced expression of energy metabolism genes in posterior cingulate neurons. Proc Natl Acad Sci USA.

[263] Cho DH, Nakamura T, Fang J, Cieplak P, Godzik A, Gu Z, Lipton SA (2009). S-nitrosylation of Drp1 mediates beta-amyloid-related mitochondrial fission and neuronal injury. Science.

[264] Lin MK, Farrer MJ (2014). Genetics and genomics of Parkinson's disease. Genome Med.

[265] Tanaka A (2010). Parkin-mediated selective mitochondrial autophagy, mitophagy: Parkin purges damaged organelles from the vital mitochondrial network. FEBS Lett.

[266] Tsai PI, Lin CH, Hsieh CH, Papakyrikos AM, Kim MJ, Napolioni V, Schoor C, Couthouis J, Wu RM, Wszolek ZK (2018). PINK1 Phosphorylates MIC60/mitofilin to control structural plasticity of mitochondrial crista junctions. Mol Cell.

[267] Yao D, Gu Z, Nakamura T, Shi ZQ, Ma Y, Gaston B, Palmer LA, Rockenstein EM, Zhang Z, Masliah E (2004). Nitrosative stress linked to sporadic Parkinson's disease: S-nitrosylation of parkin regulates its E3 ubiquitin ligase activity. Proc Natl Acad Sci USA.

[268] Zhang Z, Liu L, Jiang X, Zhai S, Xing D (2016). The essential role of Drp1 and its regulation by S-nitrosylation of Parkin in dopaminergic neurodegeneration: implications for Parkinson's disease. Antioxid Redox Signal.

[269] Oh CK, Sultan A, Platzer J, Dolatabadi N, Soldner F, McClatchy DB, Diedrich JK, Yates JR, Ambasudhan R, Nakamura T (2017). S-Nitrosylation of PINK1 attenuates PINK1/Parkin-dependent mitophagy in hiPSC-based Parkinson's disease models. Cell Rep.

[270] Cui L, Jeong H, Borovecki F, Parkhurst CN, Tanese N, Krainc D (2006). Transcriptional repression of PGC-1alpha by mutant huntingtin leads to mitochondrial dysfunction and neurodegeneration. Cell.

[271] Haun F, Nakamura T, Shiu AD, Cho DH, Tsunemi T, Holland EA, La Spada AR, Lipton SA (2013). S-nitrosylation of dynamin-related protein 1 mediates mutant huntingtin-induced mitochondrial fragmentation and neuronal injury in Huntington's disease. Antioxid Redox Signal.

[272] Wang H, Lim PJ, Karbowski M, Monteiro MJ (2009). Effects of overexpression of huntingtin proteins on mitochondrial integrity. Hum Mol Genet.

[273] Franco-Iborra S, Plaza-Zabala A, Montpeyo M, Sebastian D, Vila M, Martinez-Vicente M (2020). Mutant HTT (huntingtin) impairs mitophagy in a cellular model of Huntington disease.

[274] Nakamura T, Lipton SA (2017). 'SNO'-storms compromise protein activity and mitochondrial metabolism in neurodegenerative disorders. Trends Endocrinol Metab.

[275] Cenini G, Lloret A, Cascella R (2019). Oxidative stress in neurodegenerative diseases: from a mitochondrial point of view. Oxid Med Cell Longev.

[276] Nakamura T, Tu S, Akhtar MW, Sunico CR, Okamoto S, Lipton SA (2013). Aberrant protein s-nitrosylation in neurodegenerative diseases. Neuron.

[277] Ischiropoulos H (2009). Protein tyrosine nitration—an update. Arch Biochem Biophys.

[278] Chouchani ET, Hurd TR, Nadtochiy SM, Brookes PS, Fearnley IM, Lilley KS, Smith RA, Murphy MP (2010). Identification of S-nitrosated mitochondrial proteins by S-nitrosothiol difference in gel electrophoresis (SNO-DIGE): implications for the regulation of mitochondrial function by reversible S-nitrosation. Biochem J.

[279] Doulias PT, Tenopoulou M, Greene JL, Raju K, Ischiropoulos H (2013). Nitric oxide regulates mitochondrial fatty acid metabolism through reversible protein S-nitrosylation. Sci Signal.

[280] Bak DW, Pizzagalli MD, Weerapana E (2017). Identifying functional cysteine residues in the mitochondria. ACS Chem Biol.

[281] Gupta KJ, Shah JK, Brotman Y, Jahnke K, Willmitzer L, Kaiser WM, Bauwe H, Igamberdiev AU (2012). Inhibition of aconitase by nitric oxide leads to induction of the alternative oxidase and to a shift of metabolism towards biosynthesis of amino acids. J Exp Bot.

[282] Nimmo GA, Nimmo HG (1984). The regulatory properties of isocitrate dehydrogenase kinase and isocitrate dehydrogenase phosphatase from Escherichia coli ML308 and the roles of these activities in the control of isocitrate dehydrogenase. Eur J Biochem.

[283] Borutaite V, Budriunaite A, Brown GC (2000). Reversal of nitric oxide-, peroxynitrite- and S-nitrosothiol-induced inhibition of mitochondrial respiration or complex I activity by light and thiols. Biochim Biophys Acta.

[284] Clementi E, Brown GC, Feelisch M, Moncada S (1998). Persistent inhibition of cell respiration by nitric oxide: crucial role of S-nitrosylation of mitochondrial complex I and protective action of glutathione. Proc Natl Acad Sci USA.

[285] Galkin A, Moncada S (2007). S-nitrosation of mitochondrial complex I depends on its structural conformation. J Biol Chem.

[286] Zhang J, Jin B, Li L, Block ER, Patel JM (2005). Nitric oxide-induced persistent inhibition and nitrosylation of active site cysteine residues of mitochondrial cytochrome-c oxidase in lung endothelial cells. Am J Physiol Cell Physiol.

[287] Sun J, Morgan M, Shen RF, Steenbergen C, Murphy E (2007). Preconditioning results in S-nitrosylation of proteins involved in regulation of mitochondrial energetics and calcium transport. Circ Res.

[288] Cassina A, Radi R (1996). Differential inhibitory action of nitric oxide and peroxynitrite on mitochondrial electron transport. Arch Biochem Biophys.

[289] Castro L, Demicheli V, Tortora V, Radi R (2011). Mitochondrial protein tyrosine nitration. Free Radic Res.

[290] Ryan SD, Dolatabadi N, Chan SF, Zhang X, Akhtar MW, Parker J, Soldner F, Sunico CR, Nagar S, Talantova M (2013). Isogenic human iPSC Parkinson's model shows nitrosative stress-induced dysfunction in MEF2-PGC1alpha transcription. Cell.

[291] Okamoto S, Nakamura T, Cieplak P, Chan SF, Kalashnikova E, Liao L, Saleem S, Han X, Clemente A, Nutter A (2014). S-nitrosylation-mediated redox transcriptional switch modulates neurogenesis and neuronal cell death. Cell Rep.

[292] Rossetti ZL, Sotgiu A, Sharp DE, Hadjiconstantinou M, Neff NH (1988). 1-Methyl-4-phenyl-1,2,3,6-tetrahydropyridine (MPTP) and free radicals in vitro. Biochem Pharmacol.

[293] Sipos I, Tretter L, Adam-Vizi V (2003). Quantitative relationship between inhibition of respiratory complexes and formation of reactive oxygen species in isolated nerve terminals. J Neurochem.

[294] Cassarino DS, Fall CP, Swerdlow RH, Smith TS, Halvorsen EM, Miller SW, Parks JP, Parker WD, Bennett JP (1997). Elevated reactive oxygen species and antioxidant enzyme activities in animal and cellular models of Parkinson's disease. Biochim Biophys Acta.

[295] Calabrese V, Mancuso C, Calvani M, Rizzarelli E, Butterfield DA, Stella AM (2007). Nitric oxide in the central nervous system: neuroprotection versus neurotoxicity. Nat Rev Neurosci.

[296] Li MD, Burns TC, Morgan AA, Khatri P (2014). Integrated multi-cohort transcriptional meta-analysis of neurodegenerative diseases. Acta Neuropathol Commun.

[297] Mattson MP (2012). Energy intake and exercise as determinants of brain health and vulnerability to injury and disease. Cell Metab.

[298] Amato S, Man HY (2011). Bioenergy sensing in the brain: the role of AMP-activated protein kinase in neuronal metabolism, development and neurological diseases. Cell Cycle.

[299] Onyango IG, Lu J, Rodova M, Lezi E, Crafter AB, Swerdlow RH (2010). Regulation of neuron mitochondrial biogenesis and relevance to brain health. Biochim Biophys Acta.

[300] Palacios OM, Carmona JJ, Michan S, Chen KY, Manabe Y, Ward JL, Goodyear LJ, Tong Q (2009). Diet and exercise signals regulate SIRT3 and activate AMPK and PGC-1alpha in skeletal muscle. Aging (Albany NY).

[301] Liu L, Li Y, Wang J, Zhang D, Wu H, Li W, Wei H, Ta N, Fan Y, Liu Y (2021). Mitophagy receptor FUNDC1 is regulated by PGC-1alpha/NRF1 to fine tune mitochondrial homeostasis. EMBO Rep.

[302] Vainshtein A, Desjardins EM, Armani A, Sandri M, Hood DA (2015). PGC-1alpha modulates denervation-induced mitophagy in skeletal muscle. Skelet Muscle.

[303] Sheng B, Wang X, Su B, Lee HG, Casadesus G, Perry G, Zhu X (2012). Impaired mitochondrial biogenesis contributes to mitochondrial dysfunction in Alzheimer's disease. J Neurochem.

[304] Robinson A, Grosgen S, Mett J, Zimmer VC, Haupenthal VJ, Hundsdorfer B, Stahlmann CP, Slobodskoy Y, Muller UC, Hartmann T (2014). Upregulation of PGC-1alpha expression by Alzheimer's disease-associated pathway: presenilin 1/amyloid precursor protein (APP)/intracellular domain of APP. Aging Cell.

[305] Qin W, Haroutunian V, Katsel P, Cardozo CP, Ho L, Buxbaum JD, Pasinetti GM (2009). PGC-1alpha expression decreases in the Alzheimer disease brain as a function of dementia. Arch Neurol.

[306] Zheng B, Liao Z, Locascio JJ, Lesniak KA, Roderick SS, Watt ML, Eklund AC, Zhang-James Y, Kim PD, Hauser MA (2010). PGC-1alpha, a potential therapeutic target for early intervention in Parkinson's disease. Sci Transl Med.

[307] Ciron C, Zheng L, Bobela W, Knott GW, Leone TC, Kelly DP, Schneider BL (2015). PGC-1alpha activity in nigral dopamine neurons determines vulnerability to alpha-synuclein. Acta Neuropathol Commun.

[308] St-Pierre J, Drori S, Uldry M, Silvaggi JM, Rhee J, Jager S, Handschin C, Zheng K, Lin J, Yang W (2006). Suppression of reactive oxygen species and neurodegeneration by the PGC-1 transcriptional coactivators. Cell.

[309] Wareski P, Vaarmann A, Choubey V, Safiulina D, Liiv J, Kuum M, Kaasik A (2009). PGC-1{alpha} and PGC-1{beta} regulate mitochondrial density in neurons. J Biol Chem.

[310] Shin JH, Ko HS, Kang H, Lee Y, Lee YI, Pletinkova O, Troconso JC, Dawson VL, Dawson TM (2011). PARIS (ZNF746) repression of PGC-1alpha contributes to neurodegeneration in Parkinson's disease. Cell.

[311] Leone TC, Lehman JJ, Finck BN, Schaeffer PJ, Wende AR, Boudina S, Courtois M, Wozniak DF, Sambandam N, Bernal-Mizrachi C (2005). PGC-1alpha deficiency causes multi-system energy metabolic derangements: muscle dysfunction, abnormal weight control and hepatic steatosis. PLoS Biol.

[312] Lin J, Wu PH, Tarr PT, Lindenberg KS, St-Pierre J, Zhang CY, Mootha VK, Jager S, Vianna CR, Reznick RM (2004). Defects in adaptive energy metabolism with CNS-linked hyperactivity in PGC-1alpha null mice. Cell.

[313] Tsunemi T, Ashe TD, Morrison BE, Soriano KR, Au J, Roque RA, Lazarowski ER, Damian VA, Masliah E, La Spada AR (2012). PGC-1alpha rescues Huntington's disease proteotoxicity by preventing oxidative stress and promoting TFEB function. Sci Transl Med.

[314] Chaturvedi RK, Calingasan NY, Yang L, Hennessey T, Johri A, Beal MF (2010). Impairment of PGC-1alpha expression, neuropathology and hepatic steatosis in a transgenic mouse model of Huntington's disease following chronic energy deprivation. Hum Mol Genet.

[315] Banerjee K, Munshi S, Frank DE, Gibson GE (2015). Abnormal glucose metabolism in Alzheimer's disease: relation to autophagy/mitophagy and therapeutic approaches. Neurochem Res.

[316] Arvanitakis Z, Wilson RS, Bienias JL, Evans DA, Bennett DA (2004). Diabetes mellitus and risk of Alzheimer disease and decline in cognitive function. Arch Neurol.

[317] Hu G, Jousilahti P, Bidel S, Antikainen R, Tuomilehto J (2007). Type 2 diabetes and the risk of Parkinson's disease. Diabetes Care.

[318] Santiago JA, Potashkin JA (2013). Shared dysregulated pathways lead to Parkinson's disease and diabetes. Trends Mol Med.

[319] Mattson MP (2015). Lifelong brain health is a lifelong challenge: from evolutionary principles to empirical evidence. Ageing Res Rev.

[320] Xiromerisiou G, Hadjigeorgiou GM, Papadimitriou A, Katsarogiannis E, Gourbali V, Singleton AB (2008). Association between AKT1 gene and Parkinson's disease: a protective haplotype. Neurosci Lett.

[321] Kwok JB, Hallupp M, Loy CT, Chan DK, Woo J, Mellick GD, Buchanan DD, Silburn PA, Halliday GM, Schofield PR (2005). GSK3B polymorphisms alter transcription and splicing in Parkinson's disease. Ann Neurol.

[322] Harr SD, Simonian NA, Hyman BT (1995). Functional alterations in Alzheimer's disease: decreased glucose transporter 3 immunoreactivity in the perforant pathway terminal zone. J Neuropathol Exp Neurol.

[323] Simpson IA, Vannucci SJ, Maher F (1994). Glucose transporters in mammalian brain. Biochem Soc Trans.

[324] Landau SM, Harvey D, Madison CM, Reiman EM, Foster NL, Aisen PS, Petersen RC, Shaw LM, Trojanowski JQ, Jack CR, Jr.et al, (2010). Comparing predictors of conversion and decline in mild cognitive impairment. Neurology.

[325] Winkler EA, Nishida Y, Sagare AP, Rege SV, Bell RD, Perlmutter D, Sengillo JD, Hillman S, Kong P, Nelson AR (2015). GLUT1 reductions exacerbate Alzheimer's disease vasculo-neuronal dysfunction and degeneration. Nat Neurosci.

[326] Ruegsegger GN, Manjunatha S, Summer P, Gopala S, Zabeilski P, Dasari S, Vanderboom PM, Lanza IR, Klaus KA, Nair KS (2019). Insulin deficiency and intranasal insulin alter brain mitochondrial function: a potential factor for dementia in diabetes. FASEB J.

[327] Palombo E, Porrino LJ, Bankiewicz KS, Crane AM, Sokoloff L, Kopin IJ (1990). Local cerebral glucose utilization in monkeys with hemiparkinsonism induced by intracarotid infusion of the neurotoxin MPTP. J Neurosci Off J Soc Neurosci.

[328] Schwartzman RJ, Alexander GM (1985). Changes in the local cerebral metabolic rate for glucose in the 1-methyl-4-phenyl-1,2,3,6-tetrahydropyridine (MPTP) primate model of Parkinson's disease. Brain Res.

[329] Schwartzman RJ, Alexander GM (1987). Changes in the local cerebral metabolic rate for glucose in the MPTP primate model of Parkinson's disease. Adv Neurol.

[330] Malagelada C, Jin ZH, Greene LA (2008). RTP801 is induced in Parkinson's disease and mediates neuron death by inhibiting Akt phosphorylation/activation. J Neurosci: Off J Soc Neurosci.

[331] Murata H, Sakaguchi M, Jin Y, Sakaguchi Y, Futami J, Yamada H, Kataoka K, Huh NH (2011). A new cytosolic pathway from a Parkinson disease-associated kinase, BRPK/PINK1: activation of AKT via mTORC2. J Biol Chem.

[332] Tasaki Y, Omura T, Yamada T, Ohkubo T, Suno M, Iida S, Sakaguchi T, Asari M, Shimizu K, Matsubara K (2010). Meloxicam protects cell damage from 1-methyl-4-phenyl pyridinium toxicity via the phosphatidylinositol 3-kinase/Akt pathway in human dopaminergic neuroblastoma SH-SY5Y cells. Brain Res.

[333] Timmons S, Coakley MF, Moloney AM, C ON, (2009). Akt signal transduction dysfunction in Parkinson's disease. Neurosci Lett.

[334] Greene LA, Levy O, Malagelada C (2011). Akt as a victim, villain and potential hero in Parkinson's disease pathophysiology and treatment. Cell Mol Neurobiol.

[335] Isaev NK, Stel'mashuk EV, Zorov DB (2007). Cellular mechanisms of brain hypoglycemia. Biochemistry (Mosc).

[336] Guatteo E, Marinelli S, Geracitano R, Tozzi A, Federici M, Bernardi G, Mercuri NB (2005). Dopamine-containing neurons are silenced by energy deprivation: a defensive response or beginning of cell death?. Neurotoxicology.

[337] Bellucci A, Collo G, Sarnico I, Battistin L, Missale C, Spano P (2008). Alpha-synuclein aggregation and cell death triggered by energy deprivation and dopamine overload are counteracted by D2/D3 receptor activation. J Neurochem.

[338] Ciarmiello A, Cannella M, Lastoria S, Simonelli M, Frati L, Rubinsztein DC, Squitieri F (2006). Brain white-matter volume loss and glucose hypometabolism precede the clinical symptoms of Huntington's disease. J Nucl Med.

[339] Gamberino WC, Brennan WA (1994). Glucose transporter isoform expression in Huntington's disease brain. J Neurochem.

[340] McClory H, Williams D, Sapp E, Gatune LW, Wang P, DiFiglia M, Li X (2014). Glucose transporter 3 is a rab11-dependent trafficking cargo and its transport to the cell surface is reduced in neurons of CAG140 Huntington's disease mice. Acta Neuropathol Commun.

[341] Antonini A, Leenders KL, Spiegel R, Meier D, Vontobel P, Weigell-Weber M, Sanchez-Pernaute R, de Yebenez JG, Boesiger P, Weindl A (1996). Striatal glucose metabolism and dopamine D2 receptor binding in asymptomatic gene carriers and patients with Huntington's disease. Brain.

[342] Grafton ST, Mazziotta JC, Pahl JJ, St George-Hyslop P, Haines JL, Gusella J, Hoffman JM, Baxter LR, Phelps ME (1992). Serial changes of cerebral glucose metabolism and caudate size in persons at risk for Huntington's disease. Arch Neurol.

[343] Mazziotta JC, Phelps ME, Pahl JJ, Huang SC, Baxter LR, Riege WH, Hoffman JM, Kuhl DE, Lanto AB, Wapenski JA (1987). Reduced cerebral glucose metabolism in asymptomatic subjects at risk for Huntington's disease. N Engl J Med.

[344] Besson MT, Alegria K, Garrido-Gerter P, Barros LF, Lievens JC (2015). Enhanced neuronal glucose transporter expression reveals metabolic choice in a HD Drosophila model. PLoS ONE.

[345] Vittori A, Breda C, Repici M, Orth M, Roos RA, Outeiro TF, Giorgini F, Hollox EJ, RiotEHsD N (2014). Copy-number variation of the neuronal glucose transporter gene SLC2A3 and age of onset in Huntington's disease. Hum Mol Genet.

[346] Liu YJ, Chern Y (2015). AMPK-mediated regulation of neuronal metabolism and function in brain diseases. J Neurogenet.

[347] Ronnett GV, Ramamurthy S, Kleman AM, Landree LE, Aja S (2009). AMPK in the brain: its roles in energy balance and neuroprotection. J Neurochem.

[348] Canto C, Auwerx J (2010). AMP-activated protein kinase and its downstream transcriptional pathways. Cell Mol Life Sci: CMLS.

[349] Jager S, Handschin C, St-Pierre J, Spiegelman BM (2007). AMP-activated protein kinase (AMPK) action in skeletal muscle via direct phosphorylation of PGC-1alpha. Proc Natl Acad Sci USA.

[350] Yu L, Yang SJ (2010). AMP-activated protein kinase mediates activity-dependent regulation of peroxisome proliferator-activated receptor gamma coactivator-1alpha and nuclear respiratory factor 1 expression in rat visual cortical neurons. Neuroscience.

[351] Zong H, Ren JM, Young LH, Pypaert M, Mu J, Birnbaum MJ, Shulman GI (2002). AMP kinase is required for mitochondrial biogenesis in skeletal muscle in response to chronic energy deprivation. Proc Natl Acad Sci USA.

[352] Chaturvedi RK, Flint Beal M (2013). Mitochondrial diseases of the brain. Free Radic Biol Med.

[353] Federico A, Cardaioli E, Da Pozzo P, Formichi P, Gallus GN, Radi E (2012). Mitochondria, oxidative stress and neurodegeneration. J Neurol Sci.

[354] Ju TC, Chen HM, Chen YC, Chang CP, Chang C, Chern Y (2014). AMPK-alpha1 functions downstream of oxidative stress to mediate neuronal atrophy in Huntington's disease. Biochim Biophys Acta.

[355] Mairet-Coello G, Courchet J, Pieraut S, Courchet V, Maximov A, Polleux F (2013). The CAMKK2-AMPK kinase pathway mediates the synaptotoxic effects of Abeta oligomers through Tau phosphorylation. Neuron.

[356] Son SM, Jung ES, Shin HJ, Byun J, Mook-Jung I (2012). Abeta-induced formation of autophagosomes is mediated by RAGE-CaMKKbeta-AMPK signaling. Neurobiol Aging.

[357] Du H, Guo L, Yan S, Sosunov AA, McKhann GM, Yan SS (2010). Early deficits in synaptic mitochondria in an Alzheimer's disease mouse model. Proc Natl Acad Sci USA.

[358] Manczak M, Anekonda TS, Henson E, Park BS, Quinn J, Reddy PH (2006). Mitochondria are a direct site of A beta accumulation in Alzheimer's disease neurons: implications for free radical generation and oxidative damage in disease progression. Hum Mol Genet.

[359] Calkins MJ, Reddy PH (2011). Assessment of newly synthesized mitochondrial DNA using BrdU labeling in primary neurons from Alzheimer's disease mice: Implications for impaired mitochondrial biogenesis and synaptic damage. Biochim Biophys Acta.

[360] Thornton C, Bright NJ, Sastre M, Muckett PJ, Carling D (2011). AMP-activated protein kinase (AMPK) is a tau kinase, activated in response to amyloid beta-peptide exposure. Biochem J.

[361] Abramov AY, Canevari L, Duchen MR (2004). Calcium signals induced by amyloid beta peptide and their consequences in neurons and astrocytes in culture. Biochim Biophys Acta.

[362] Shimohama S, Tanino H, Kawakami N, Okamura N, Kodama H, Yamaguchi T, Hayakawa T, Nunomura A, Chiba S, Perry G (2000). Activation of NADPH oxidase in Alzheimer's disease brains. Biochem Biophys Res Commun.

[363] Vingtdeux V, Davies P, Dickson DW, Marambaud P (2011). AMPK is abnormally activated in tangle- and pre-tangle-bearing neurons in Alzheimer's disease and other tauopathies. Acta Neuropathol.

[364] Ma T, Chen Y, Vingtdeux V, Zhao H, Viollet B, Marambaud P, Klann E (2014). Inhibition of AMP-activated protein kinase signaling alleviates impairments in hippocampal synaptic plasticity induced by amyloid beta. J Neurosci Off J Soc Neurosci.

[365] Chen Y, Zhou K, Wang R, Liu Y, Kwak YD, Ma T, Thompson RC, Zhao Y, Smith L, Gasparini L (2009). Antidiabetic drug metformin (GlucophageR) increases biogenesis of Alzheimer's amyloid peptides via up-regulating BACE1 transcription. Proc Natl Acad Sci USA.

[366] DiTacchio KA, Heinemann SF, Dziewczapolski G (2015). Metformin treatment alters memory function in a mouse model of Alzheimer's disease. J Alzheimers Dis.

[367] Park H, Kam TI, Kim Y, Choi H, Gwon Y, Kim C, Koh JY, Jung YK (2012). Neuropathogenic role of adenylate kinase-1 in Abeta-mediated tau phosphorylation via AMPK and GSK3beta. Hum Mol Genet.

[368] Won JS, Im YB, Kim J, Singh AK, Singh I (2010). Involvement of AMP-activated-protein-kinase (AMPK) in neuronal amyloidogenesis. Biochem Biophys Res Commun.

[369] Greco SJ, Hamzelou A, Johnston JM, Smith MA, Ashford JW, Tezapsidis N (2011). Leptin boosts cellular metabolism by activating AMPK and the sirtuins to reduce tau phosphorylation and beta-amyloid in neurons. Biochem Biophys Res Commun.

[370] Greco SJ, Sarkar S, Johnston JM, Tezapsidis N (2009). Leptin regulates tau phosphorylation and amyloid through AMPK in neuronal cells. Biochem Biophys Res Commun.

[371] Chiang MC, Cheng YC, Chen SJ, Yen CH, Huang RN (2016). Metformin activation of AMPK-dependent pathways is neuroprotective in human neural stem cells against Amyloid-beta-induced mitochondrial dysfunction. Exp Cell Res.

[372] Vingtdeux V, Giliberto L, Zhao H, Chandakkar P, Wu Q, Simon JE, Janle EM, Lobo J, Ferruzzi MG, Davies P (2010). AMP-activated protein kinase signaling activation by resveratrol modulates amyloid-beta peptide metabolism. J Biol Chem.

[373] Wang ZG, Yang C, Zhu B, Hua F (2015). AMPK-dependent autophagic activation is probably involved in the mechanism of resveratrol exerting therapeutic effects for Alzheimer's disease. Rejuvenation Res.

[374] Hang L, Thundyil J, Lim KL (2015). Mitochondrial dysfunction and Parkinson disease: a Parkin-AMPK alliance in neuroprotection. Ann NY Acad Sci.

[375] Choi JS, Park C, Jeong JW (2010). AMP-activated protein kinase is activated in Parkinson's disease models mediated by 1-methyl-4-phenyl-1,2,3,6-tetrahydropyridine. Biochem Biophys Res Commun.

[376] Horvath TL, Erion DM, Elsworth JD, Roth RH, Shulman GI, Andrews ZB (2011). GPA protects the nigrostriatal dopamine system by enhancing mitochondrial function. Neurobiol Dis.

[377] Kim TW, Cho HM, Choi SY, Suguira Y, Hayasaka T, Setou M, Koh HC, Hwang EM, Park JY, Kang SJ (2013). (ADP-ribose) polymerase 1 and AMP-activated protein kinase mediate progressive dopaminergic neuronal degeneration in a mouse model of Parkinson's disease. Cell Death Dis.

[378] Chou SY, Lee YC, Chen HM, Chiang MC, Lai HL, Chang HH, Wu YC, Sun CN, Chien CL, Lin YS (2005). CGS21680 attenuates symptoms of Huntington's disease in a transgenic mouse model. J Neurochem.

[379] Ju TC, Chen HM, Lin JT, Chang CP, Chang WC, Kang JJ, Sun CP, Tao MH, Tu PH, Chang C (2011). Nuclear translocation of AMPK-alpha1 potentiates striatal neurodegeneration in Huntington's disease. J Cell Biol.

[380] Zheng J, Winderickx J, Franssens V, Liu B (2018). A mitochondria-associated oxidative stress perspective on Huntington's disease. Front Mol Neurosci.

[381] Choo YS, Johnson GV, MacDonald M, Detloff PJ, Lesort M (2004). Mutant huntingtin directly increases susceptibility of mitochondria to the calcium-induced permeability transition and cytochrome c release. Hum Mol Genet.

[382] Lim D, Fedrizzi L, Tartari M, Zuccato C, Cattaneo E, Brini M, Carafoli E (2008). Calcium homeostasis and mitochondrial dysfunction in striatal neurons of Huntington disease. J Biol Chem.

[383] Panov AV, Lund S, Greenamyre JT (2005). Ca2+-induced permeability transition in human lymphoblastoid cell mitochondria from normal and Huntington's disease individuals. Mol Cell Biochem.

[384] Solans A, Zambrano A, Rodriguez M, Barrientos A (2006). Cytotoxicity of a mutant huntingtin fragment in yeast involves early alterations in mitochondrial OXPHOS complexes II and III. Hum Mol Genet.

[385] Chang DT, Rintoul GL, Pandipati S, Reynolds IJ (2006). Mutant huntingtin aggregates impair mitochondrial movement and trafficking in cortical neurons. Neurobiol Dis.

[386] Subhramanyam CS, Wang C, Hu Q, Dheen ST (2019). Microglia-mediated neuroinflammation in neurodegenerative diseases. Semin Cell Dev Biol.

[387] van Horssen J, van Schaik P, Witte M (2019). Inflammation and mitochondrial dysfunction: a vicious circle in neurodegenerative disorders?. Neurosci Lett.

[388] Hughes V (2012). Microglia: the constant gardeners. Nature.

[389] Stence N, Waite M, Dailey ME (2001). Dynamics of microglial activation: a confocal time-lapse analysis in hippocampal slices. Glia.

[390] Brown MR, Sullivan PG, Geddes JW (2006). Synaptic mitochondria are more susceptible to Ca2+overload than nonsynaptic mitochondria. J Biol Chem.

[391] Kostuk EW, Cai J, Iacovitti L (2018). Regional microglia are transcriptionally distinct but similarly exacerbate neurodegeneration in a culture model of Parkinson's disease. J Neuroinflammation.

[392] Liemburg-Apers DC, Willems PH, Koopman WJ, Grefte S (2015). Interactions between mitochondrial reactive oxygen species and cellular glucose metabolism. Arch Toxicol.

[393] Wada J (2017). Reprogramming of metabolism in immune-mediated cells. Diabetol Int.

[394] Zhang Y, Chen K, Sloan SA, Bennett ML, Scholze AR, O'Keeffe S, Phatnani HP, Guarnieri P, Caneda C, Ruderisch N (2014). An RNA-sequencing transcriptome and splicing database of glia, neurons, and vascular cells of the cerebral cortex. J Neurosci Off J Soc Neurosci.

[395] Ghosh S, Castillo E, Frias ES, Swanson RA (2018). Bioenergetic regulation of microglia. Glia.

[396] Gimeno-Bayon J, Lopez-Lopez A, Rodriguez MJ, Mahy N (2014). Glucose pathways adaptation supports acquisition of activated microglia phenotype. J Neurosci Res.

[397] Voloboueva LA, Emery JF, Sun X, Giffard RG (2013). Inflammatory response of microglial BV-2 cells includes a glycolytic shift and is modulated by mitochondrial glucose-regulated protein 75/mortalin. FEBS Lett.

[398] Zell R, Geck P, Werdan K, Boekstegers P (1997). TNF-alpha and IL-1 alpha inhibit both pyruvate dehydrogenase activity and mitochondrial function in cardiomyocytes: evidence for primary impairment of mitochondrial function. Mol Cell Biochem.

[399] Reynolds WF, Rhees J, Maciejewski D, Paladino T, Sieburg H, Maki RA, Masliah E (1999). Myeloperoxidase polymorphism is associated with gender specific risk for Alzheimer's disease. Exp Neurol.

[400] Palomer X, Alvarez-Guardia D, Rodriguez-Calvo R, Coll T, Laguna JC, Davidson MM, Chan TO, Feldman AM, Vazquez-Carrera M (2009). TNF-alpha reduces PGC-1alpha expression through NF-kappaB and p38 MAPK leading to increased glucose oxidation in a human cardiac cell model. Cardiovasc Res.

[401] Islinger M, Voelkl A, Fahimi HD, Schrader M (2018). The peroxisome: an update on mysteries 2.0. Histochem Cell Biol.

[402] Ginsberg L, Rafique S, Xuereb JH, Rapoport SI, Gershfeld NL (1995). Disease and anatomic specificity of ethanolamine plasmalogen deficiency in Alzheimer's disease brain. Brain Res.

[403] Kou J, Kovacs GG, Hoftberger R, Kulik W, Brodde A, Forss-Petter S, Honigschnabl S, Gleiss A, Brugger B, Wanders R (2011). Peroxisomal alterations in Alzheimer's disease. Acta Neuropathol.

[404] Grimm MO, Grimm HS, Tomic I, Beyreuther K, Hartmann T, Bergmann C (2008). Independent inhibition of Alzheimer disease beta- and gamma-secretase cleavage by lowered cholesterol levels. J Biol Chem.

[405] Jo DS, Park NY, Cho DH (2020). Peroxisome quality control and dysregulated lipid metabolism in neurodegenerative diseases. Exp Mol Med.

[406] Fabelo N, Martin V, Santpere G, Marin R, Torrent L, Ferrer I, Diaz M (2011). Severe alterations in lipid composition of frontal cortex lipid rafts from Parkinson's disease and incidental Parkinson's disease. Mol Med.

[407] Brodde A, Teigler A, Brugger B, Lehmann WD, Wieland F, Berger J, Just WW (2012). Impaired neurotransmission in ether lipid-deficient nerve terminals. Hum Mol Genet.

[408] Fransen M, Lismont C, Walton P (2017). The peroxisome-mitochondria connection: how and why?. Int J Mol Sci.

[409] Lautenschager J, Kaminski Schierle GS (2019). Mitochondrial degradation of amyloidogenic proteins—a new perspective for neurodegenerative diseases. Prog Neurobiol.

[410] Lu B, Guo S (2020). Mechanisms linking mitochondrial dysfunction and proteostasis failure. Trends Cell Biol.

[411] Lambert JP, Luongo TS, Tomar D, Jadiya P, Gao E, Zhang X, Lucchese AM, Kolmetzky DW, Shah NS, Elrod JW (2019). MCUB regulates the molecular composition of the mitochondrial calcium uniporter channel to limit mitochondrial calcium overload during stress. Circulation.

[412] Csordas G, Golenar T, Seifert EL, Kamer KJ, Sancak Y, Perocchi F, Moffat C, Weaver D, Perez SF, Bogorad R (2013). MICU1 controls both the threshold and cooperative activation of the mitochondrial Ca(2)(+) uniporter. Cell Metab.

[413] Mallilankaraman K, Doonan P, Cardenas C, Chandramoorthy HC, Muller M, Miller R, Hoffman NE, Gandhirajan RK, Molgo J, Birnbaum MJ (2012). MICU1 is an essential gatekeeper for MCU-mediated mitochondrial Ca(2+) uptake that regulates cell survival. Cell.

[414] Gottschalk B, Klec C, Leitinger G, Bernhart E, Rost R, Bischof H, Madreiter-Sokolowski CT, Radulovic S, Eroglu E, Sattler W (2019). MICU1 controls cristae junction and spatially anchors mitochondrial Ca(2+) uniporter complex. Nat Commun.

[415] Patron M, Granatiero V, Espino J, Rizzuto R, De Stefani D (2019). MICU3 is a tissue-specific enhancer of mitochondrial calcium uptake. Cell Death Differ.

[416] Sancak Y, Markhard AL, Kitami T, Kovacs-Bogdan E, Kamer KJ, Udeshi ND, Carr SA, Chaudhuri D, Clapham DE, Li AA (2013). EMRE is an essential component of the mitochondrial calcium uniporter complex. Science.

[417] van der Laan M, Horvath SE, Pfanner N (2016). Mitochondrial contact site and cristae organizing system. Curr Opin Cell Biol.

[418] Gottschalk B, Klec C, Waldeck-Weiermair M, Malli R, Graier WF (2018). Intracellular Ca(2+) release decelerates mitochondrial cristae dynamics within the junctions to the endoplasmic reticulum. Pflugers Arch.

[419] Konig T, Troder SE, Bakka K, Korwitz A, Richter-Dennerlein R, Lampe PA, Patron M, Muhlmeister M, Guerrero-Castillo S, Brandt U (2016). The m-AAA protease associated with neurodegeneration limits MCU activity in mitochondria. Mol Cell.

[420] Patron M, Sprenger HG, Langer T (2018). m-AAA proteases, mitochondrial calcium homeostasis and neurodegeneration. Cell Res.

